# Assessing the Risk of Transfer of Microorganisms at the International Space Station Due to Cargo Delivery by Commercial Resupply Vehicles

**DOI:** 10.3389/fmicb.2020.566412

**Published:** 2020-11-06

**Authors:** Snehit Mhatre, Jason M. Wood, Aleksandra Checinska Sielaff, Maximilian Mora, Stefanie Duller, Nitin Kumar Singh, Fathi Karouia, Christine Moissl-Eichinger, Kasthuri Venkateswaran

**Affiliations:** ^1^Biotechnology and Planetary Protection Group, Jet Propulsion Laboratory, California Institute of Technology, Pasadena, CA, United States; ^2^Department of Internal Medicine, Medical University of Graz, Graz, Austria; ^3^Space Bioscience Division, NASA Ames Research Center, Moffett Field, CA, United States; ^4^Department of Pharmaceutical Chemistry, University of California, San Francisco, San Francisco, CA, United States; ^5^BioTechMed-Graz, Graz, Austria

**Keywords:** forward contamination, microbial diversity, commercial resupply vehicle, viability, International Space Station

## Abstract

**Background:**

With increasing numbers of interplanetary missions, there is a need to establish robust protocols to ensure the protection of extraterrestrial planets being visited from contamination by terrestrial life forms. The current study is the first report comparing the commercial resupply vehicle (CRV) microbiome with the International Space Station (ISS) microbiome to understand the risks of contamination, thus serving as a model system for future planetary missions.

**Results:**

Samples obtained from the internal surfaces and ground support equipment of three CRV missions were subjected to various molecular techniques for microbial diversity analysis. In total, 25 samples were collected with eight defined locations from each CRV mission prior to launch. In general, the internal surfaces of vehicles were clean, with an order of magnitude fewer microbes compared to ground support equipment. The first CRV mission had a larger microbial population than subsequent CRV missions, which were clean as compared to the initial CRV locations sampled. Cultivation assays showed the presence of *Actinobacteria*, *Proteobacteria*, *Firmicutes*, and *Bacteroidetes* and members of *Ascomycota* and *Basidiomycota*. As expected, shotgun metagenome analyses revealed the presence of more microbial taxa compared to cultivation-based assays. The internal locations of the CRV microbiome reportedly showed the presence of microorganisms capable of tolerating ultraviolet radiation (e.g., *Bacillus firmus*) and clustered separately from the ISS microbiome.

**Conclusions:**

The metagenome sequence comparison of the CRV microbiome with the ISS microbiome revealed significant differences showing that CRV microbiomes were a negligible part of the ISS environmental microbiome. These findings suggest that the maintenance protocols in cleaning CRV surfaces are highly effective in controlling the contaminating microbial population during cargo transfer to the ISS via the CRV route.

## Introduction

Spacefaring nations carrying out interplanetary missions are subject to rules and regulations that were designed and instigated by the Committee on Space Research (COSPAR) in 1967 (COSPAR, 2011). Protocols for cleaning and sterilization are of the highest priority and are regularly practiced and meticulously followed during spacecraft assembly for various missions (Benardini et al., 2014). Cleanrooms are where various spacecraft subsystems, including commercial resupply vehicles (CRV) transporting cargo to the International Space Station (ISS), are assembled. These cleanrooms undergo daily cleaning procedures, including vacuuming and mopping of floors with 70% Isopropanol, high efficiency particulate arrestance (HEPA) air filtration, regular replacement of sticky mats at the entrance, and strict gowning procedures. Utmost care is taken by personal entering these facilities, as they are required to put on face masks, gloves, bodysuits, shoe covers, cleanroom boots, and cover hair and beards with nets (Benardini et al., 2014). These precautionary measures are a part of routine exercises, because humans are the primary source of microbial contamination (Moissl-Eichinger et al., 2015; Checinska Sielaff et al., 2019; Avila-Herrera et al., 2020). On average, humans shed 1.5 million skin cells an hour which carry approximately 15 million bacterial cells, thus becoming a significant source of the indoor microbiome (Jaffal et al., 1997; Lax et al., 2014). Many reports showed transfer of microorganisms via direct contact to indoor surfaces like keyboards (Fierer et al., 2010), restrooms (Flores et al., 2011), offices (Hewitt et al., 2012), kitchens (Flores et al., 2013), airplanes (McManus and Kelley, 2005; Korves et al., 2013), and hospitals (Kembel et al., 2012; Lax et al., 2017). Because of the human presence in these cleanrooms, their surfaces are not expected to be free of microbial burden.

The ISS and CRV assembly cleanrooms, including spacecraft assembly facility (SAF) environments, are strictly controlled and monitored for airflow, water circulation, temperature, and humidity; however, individual differences do exist between the two systems (Checinska et al., 2015). The ISS is a closed system where the air is recirculated after purification, while cleanrooms get a constant supply of fresh air from the outside environment. Additionally, astronauts live and work on the ISS as humans do in a typical household on Earth, while SAF cleanrooms have strict requirements that avoid activities like eating or sleeping. At a given point in time, the ISS harbors around six astronauts with a maximum of around 13 crew, while approximately 50 people can work in a cleanroom every day in a highly regulated manner. Despite high human traffic in SAF, the microbial burden was minimal and ranged between 10^2^ and 10^4^ cells per m^2^ (Hendrickson et al., 2017). It has also been shown that the cleaning procedures followed at SAF significantly decreased the number of microorganisms, but these procedures led to the selection of hardy, robust microorganisms capable of surviving extreme oligotrophic conditions (Gioia et al., 2007; Vaishampayan et al., 2012; Mahnert et al., 2015). Cargos such as food and equipment for scientific experiments are assembled in clean conditions and delivered to the ISS via CRV. All spacefaring nations impose cleanroom requirements when packaging their cargos that are resupplying the ISS.

**FIGURE 1:**
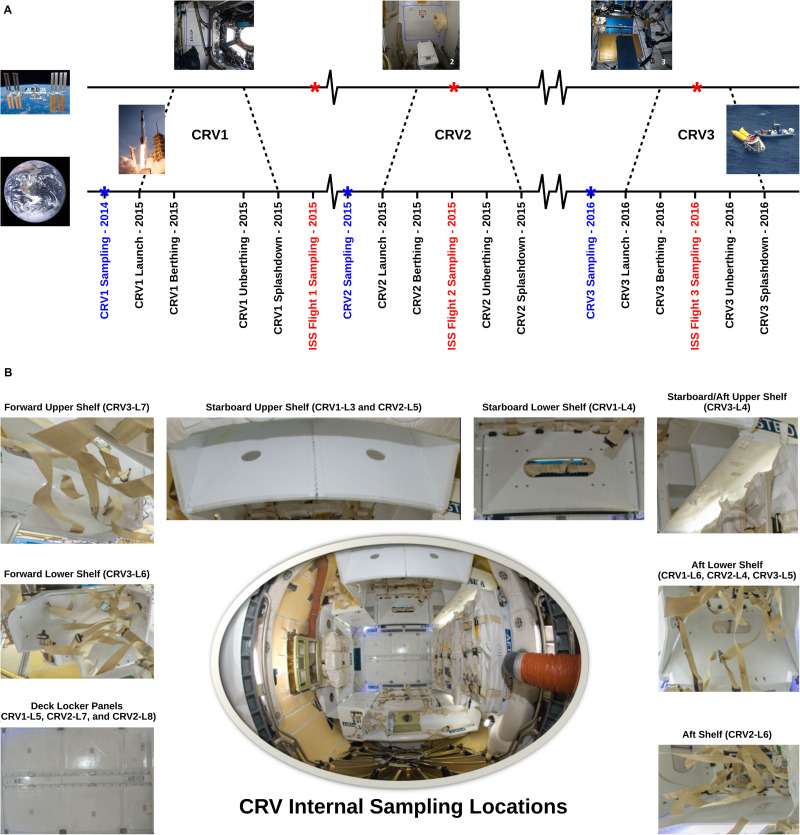
**(A)** Sampling time line for the CRV and the ISS samples. Blue labels mark sampling events conducted on CRV prior to launch, and red labels mark sampling events on board the ISS. **(B)** Representative sampling locations of internal CRV surfaces. Pictures: NASA/ESA Credit.

The objective of this study is to decipher the role of CRV systems in potentially transporting microorganisms to the ISS to understand if CRV could be a source of microbial contamination of the ISS. Sampling of various surfaces of CRV systems (CRV1, CRV2, and CRV3) was carried out prior to launch (∼1 month) and corresponding in-flight sampling of the ISS (Flight 1, Flight 2, and Flight 3) was designed to be within 25–51 days after docking of the respective CRV systems (see Figure 1 for a timeline). This sampling scheme was implemented to help understand whether CRV1 had any influence on the microbial population of ISS Flight 1, whether CRV2 had any influence on the microbial population of ISS Flight 2, or whether CRV3 had any influence on the microbial population of ISS Flight 3. Microbial burden associated with CRV environmental surface samples were estimated using traditional techniques (e.g., colony counts), and molecular techniques like adenosine triphosphate (ATP) assay and 16S rRNA gene (bacteria) and internal transcribed spacer (ITS) region (fungi) quantification using quantitative polymerase chain reaction (qPCR) assays. In addition to characterizing cultivable microbial diversity using the Sanger sequencing method, the CRV samples were more thoroughly analyzed using a shotgun metagenome sequencing approach. Subsequently, the microbial diversity of the CRV samples was compared with the ISS environmental microbiome (Checinska Sielaff et al., 2019) to understand the potential transfer of biological materials from Earth to the ISS.

## Materials and Methods

In this study, internal surfaces and external ground support equipment (GSE) of three CRV destined to the ISS were sampled (*N* = 22 and 3 controls; Table 1). Samples collected aboard the ISS were done during the berthing period of their respective vehicles, except for CRV1 (see Figure 1 for a timeline) (Checinska Sielaff et al., 2019). Eight samples were collected in 2014 for CRV1, nine in 2015 for CRV2, and eight in 2016 for CRV3. Since CRV3 vehicle surfaces were extremely clean and all analyses, including traditional microbiology assays, were below detection limits, data pertaining to CRV3 vehicle locations are not presented in this study. Since CRV were not reused during this study, we could not sample from the same capsule multiple times. Efforts were taken to collect samples as close together as possible in subsequent CRV, but due to differences in configuration of each vehicle, it was not always possible to collect samples from the same locations. All CRV sampled were manufactured by the same vendor.

**TABLE 1:** Descriptions of sampling locations on commercial resupply vehicles.

**Sample ID**	**Description**	**Category**
CRV1-L1	Ladder platform just prior to the vehicle entrance	GSE
CRV1-L2	Clean room floor near the ladder that leads up to the vehicle entrance	GSE
CRV1-L3	Upper shelf in starboard side of the vehicle	Internal
CRV1-L4	Lower shelf in starboard side of the vehicle	Internal
CRV1-L5	Front panels in deck side of the vehicle	Internal
CRV1-L6	Lower shelf in aft side of the vehicle	Internal
CRV1-L7	Ladder steps that leads up to the platform near the vehicle entrance	GSE
CRV1-FC	Control wipe exposed to the air for 30 s inside the vehicle	Control
CRV2-L1	Right leg/side of ladder	GSE
CRV2-L2	Left leg/side of ladder	GSE
CRV2-L3	Left and right rail arms of the ladder above the platform	GSE
CRV2-L4	Lower shelf in aft side of the vehicle	Internal
CRV2-L5	Upper shelf in starboard side of the vehicle	Internal
CRV2-L6	Upper shelf in aft side of the vehicle	Internal
CRV2-L7	Locker panels in deck side of the vehicle	Internal
CRV2-L8	Locker panels in deck side of the vehicle near the aft side	Internal
CRV2-FC	Control wipe exposed to the air for 30 s inside the vehicle	Control
CRV3-L1	Ladder platform just prior to the vehicle entrance	GSE
CRV3-L2	Left rail arm of the ladder above the platform	GSE
CRV3-L3	Right rail arm of the ladder above the platform	GSE
CRV3-L4	Upper shelf in starboard side of the vehicle near the aft side	Internal
CRV3-L5	Lower shelf in aft side of the vehicle	Internal
CRV3-L6	Lower shelf in forward side of the vehicle	Internal
CRV3-L7	Upper shelf in forward side of the vehicle	Internal
CRV3-FC	Control wipe exposed to the air for 30 s inside the vehicle	Control

*The nomenclature used to describe the sides of the cargo resupply vehicle are based on the following reference directions, with respect to the +X, +Y, +Z Local Vertical, Local Horizontal, and Velocity Vector. CRV, commercial resupply vehicle; GSE, ground support equipment.*

Sampling kits were assembled as detailed in our previous ISS studies (Singh et al., 2018; Checinska Sielaff et al., 2019; Avila-Herrera et al., 2020) to facilitate comparisons between these datasets. Briefly, each polyester wipe (9″ × 9″; ITW Texwipe, Mahwah, NJ, United States) was soaked in 15 mL of sterile molecular grade water (Sigma-Aldrich, St. Louis, MO, United States) for 30 min followed by transfer to a sterile zip lock bag (Venkateswaran et al., 2012). Several locations were sampled on CRV surfaces using polyester wipes, and descriptions of the locations are summarized in Table 1. A single wipe was used for each location by donning sterile gloves (KIMTEC Pure G3 White; Nitrile Clean-room Certified; Cat. HC61190; Fisher Scientific, Waltham, MA, United States) to collect a sample of one-meter square area. The samples were stored at 4°C and shipped to Jet Propulsion Laboratory (JPL, Pasadena, CA, United States) for processing. Microbiological analyses were carried out within 48 h of sampling at JPL. A control wipe (environmental control) was taken out from the zip lock bag, unfolded, waved for 30 s, and packed back inside a new sterile zip lock. One control wipe was included for each CRV sampling session. Similarly, an unused wipe that was flown to the sampling location and brought back to JPL along with the samples served as a negative control for sterility testing. If field controls (wipes that were exposed to the CRV environment but not used in active sampling) showed any signs of microbial growth, then negative controls would be assayed for cultivable counts to check sterility of the wipes used for sampling. However, none of the field controls showed any colony forming units (CFU) for all CRV sampling events.

### Sample Collection and Processing

Samples from CRV were processed in the same way as ISS samples from our previous studies (Singh et al., 2018; Checinska Sielaff et al., 2019; Avila-Herrera et al., 2020). Briefly, each wipe was transferred to an individual 500 mL bottle containing 200 mL of sterile phosphate-buffered saline (PBS; Sigma-Aldrich, St. Louis, MO, United States) and vigorously hand shaken for 2 min. The resulting suspension was then concentrated to 4 mL using a CP-150 concentration pipette (Innova Prep, Drexel, MO, United States). Both negative and field controls used in this study were processed the same way as other samples for comparison. The concentrated sample (4 mL) were further aliquoted to four different parts for shotgun metagenome analysis (2 × 1.5 mL; PMA treated and untreated), ATP analysis (900 μL), and for growing microorganisms (100 μL). Due to the measurement of different kinds of microorganisms, that require multiple culture media (3 types) in duplicates, the aliquots kept for growing microorganisms were further diluted (10^–1^ and 10^–2^) and used.

### Culture Based Microbial Diversity

Due to proprietary cleaning regimes, the surfaces of CRV might be oligotrophic. Although most nutrient and carbon sources necessary for microbial growth are presumably lacking or at very-low concentrations, these surfaces may still harbor microorganisms that are either dormant or slowly metabolizing. Furthermore, microorganisms adapted to more stringent conditions may not necessarily grow on normal nutrient media. Therefore, to target the slow-growing microbial population, Reasoner’s 2A agar (R2A; Difco, MI, United States) media (Reasoner and Geldreich, 1985) was used. Similarly, as humans are always near the CRV during the assembly and packaging processes, potential opportunistic pathogenic microorganisms may transfer to the CRV surfaces. As a result, the CRV samples were also plated on blood agar media (BA; Difco, MI, United States) to decipher the potentially pathogenic microbial population. Additionally, the samples were also plated on potato dextrose agar (PDA; Difco, MI, United States) medium, thus targeting the fungal population on CRV.

To estimate the bacterial and fungal populations, the concentrated sample was diluted to a suitable serial 10-fold dilution in sterile PBS. A volume of 100 μl of suspension from the serially diluted sample was spread onto R2A media plates and PDA plates with chloramphenicol (100 μg/mL; Sigma-Aldrich, St. Louis, MO, United States), incubated at 25°C for 7 days for bacterial and fungal population enumeration, respectively. For enumeration of human commensal microorganisms, 100 μl of serially diluted sample was also spread onto BA and incubated at 35°C for 2 days. Distinct isolated colonies were transferred to fresh media and subsequently archived in semi-solid R2A media (dilution 1:10) and stored at room temperature.

### Strain Identification

Bacterial isolates were identified by amplifying partial 16S rRNA genes using the primers 9bF (5′- GRGTTTGATCCTGG CTCAG-3′) (Huber et al., 2002) and 1406uR (5′- ACGGG CGGTGTGTRCAA-3′) (Lane, 1991) and the following cycling conditions: initial denaturation at 95°C for 2 min, 10 cycles of denaturing at 96°C for 30 s, annealing at 60°C for 30 s and elongation at 72°C for 60 s, followed by another 25 cycles of denaturing at 94°C for 30 s, annealing at 60°C for 30 s and elongation at 72°C for 60 s and a final elongation step at 72°C for 10 min. The template was either a small fraction of a picked colony in a colony-PCR assay or 5–20 ng of DNA purified from culture via the peqGOLD Bacterial DNA Kit (peqlab, Germany). The 16S rRNA gene amplicons were Sanger-sequenced (Eurofins, Germany) and the obtained sequences were classified using the EzTaxon identification service (Yoon et al., 2017).

Fungal isolates were identified by amplifying their ITS region using the primers ITS1F (5′-CTTGGTCATTTAGAGG AAGTAA-3′) and ITS4 (5′-TCCTCCGCTTATTGATATGC-3′) (Manter and Vivanco, 2007) at the following cycling conditions: initial denaturation at 95°C for 10 min, followed by 35 cycles of denaturing at 94°C for 60 s, annealing at 51°C for 60 s, elongation at 72°C for 60 s, and a final elongation step at 72°C for 8 min. The amplicons were Sanger-sequenced (Eurofins, Germany) and the obtained sequences were classified using the curated databases UNITE version 7.2 (Kõljalg et al., 2013) and BOLD version 4 (Ratnasingham and Hebert, 2013).

### Molecular Methods for Quantitation of Total and Viable Microorganisms

#### ATP Assay

To determine the total and intracellular ATP from all samples, a bioluminescence assay was performed using the CheckLite HS Kit (Kikkoman, Japan) and the manufacturer’s protocol, as described previously (Venkateswaran et al., 2003). For total ATP assay (dead and viable microbes), four replicates of 100 μl sample were mixed with 100 μl of benzalkonium chloride, a cell lysing reagent and incubated at room temperature for 1 min. To this mixture, 100 μl of luciferin-luciferase reagent was added, and the resulting bioluminescence was measured immediately using a Lumitester K-210 luminometer (Kikkoman, Japan). For intracellular ATP (viable microorganisms), 500 μl of the serially diluted sample was amended with a 50 μl of an ATP-eliminating reagent (apyrase, adenosine deaminase) and incubated for 30 min at room temperature for eliminating extracellular ATP. Following which, the ATP assay was performed as described previously for the total ATP. It has been reported that one relative luminescent unit (RLU), the unit of measurement of ATP, was considered equivalent to approximately one CFU (La Duc et al., 2004).

#### PMA-Viability Assay

Before DNA extraction, half of the sample was treated with propidium monoazide (PMA; Biotium, Inc., Hayward, CA, United States) so that the microbiome of intact/viable cells (PMA treatment) could be characterized. The PMA-untreated samples yielded information about the total microbial population (including free DNA, dead cells, cells with a compromised cell membrane, intact cells, and viable cells). PMA binds to DNA, making the DNA unavailable for amplification during PCR steps (Nocker et al., 2007). Due to its higher molecular weight and/or charge, PMA cannot penetrate cells that have an intact cell membrane (i.e., viable) but can bind to free floating DNA or DNA inside cells with a compromised cell membrane (i.e., dead cells) (Nocker et al., 2006, 2007). It is in this way that many studies have utilized PMA to distinguish between intact/viable cells and compromised/dead cells (Lin et al., 2011; Vaishampayan et al., 2013; Checinska et al., 2015; Jäger et al., 2018; Singh et al., 2018; Checinska Sielaff et al., 2019; Avila-Herrera et al., 2020).

A 3 mL aliquot of the concentrated sample was split into two halves. One 1.5 mL aliquot was treated with PMA (Biotium, Inc., Hayward, CA, United States) to a final concentration of 25 μM, followed by 5 min incubation in the dark at room temperature. The sample was then exposed to photoactivation for 15 min in a PMA-Lite LED Photolysis Device (Biotium, Inc., Hayward, CA, United States). The other aliquot with no PMA treatment was also incubated in dark for 5 min followed by photoactivation for 15 min like the PMA treated aliquot. Both PMA treated and untreated samples were then each split into half again. One half (750 μl) of each sample was then transferred to Lysing Matrix E tube (MP Biomedicals, Santa Ana, CA, United States) and subjected to bead beating for 60 s to allow cell disruption of hardy cells and spores with a limited loss of microbial diversity. The mechanically disrupted sample was then mixed with the unprocessed counterpart and used for DNA extraction via the Maxwell 16 automated system (Promega, Madison, WI, United States), per the manufacturer’s protocol. DNA was extracted in 50 μl volume and stored at −20°C until further processing.

#### Quantitative PCR Assay

Real-time quantitative polymerase chain reaction (qPCR) assays, targeting the 16S rRNA gene (bacteria) and ITS region (fungi), were performed in triplicate with a SmartCycler (Cepheid, CA, United States) to quantify the bacterial and fungal burden. The following primers were used for targeting the 16S rRNA gene: 1369F (5′-CGGTGAATACGTTCYCGG-3′) and modified 1492R (5′-GGWTACCTTGTTACGACTT-3′) were used for this analysis (Suzuki et al., 2000). Primers targeting the ITS region, were NS91 (5′-GTCCCTGCCCTTTGTACACAC-3′) and ITS51 (5′-ACCTTGTTACGACTTTTACTTCCTC-3′) (Onofri et al., 2012). Each 25 μL reaction consisted of 12.5 μL of 2X iQ SYBR Green Supermix (Bio-Rad, Hercules, CA, United States), 1 μL each of forward and reverse oligonucleotide primers (10 μM each), and 1 μL of template DNA. Purified DNA from Model Microbial Consortium (Kwan et al., 2011) served as the positive control and DNase/RNase free molecular-grade distilled water (Promega, Madison, WI, United States) was used as the negative control. These controls were included in all qPCR runs. The reaction conditions were as follows: a 3 min denaturation at 95°C, followed by 40 cycles of denaturation at 95°C for 15 s, and combined annealing and extension at 55°C for 35 s. The number of gene copies were determined from the standard curve. The 16S rRNA gene of *Bacillus pumilus* SAFR-032 and the ITS region of *Aureobasidium pullulans* 28v1 were synthesized and used for preparing standard curves as described previously (Checinska et al., 2015). The qPCR efficiency was ∼98% for each run. Negative controls yielded similar values (∼100 copies) despite using either 1 or 10 μL of DNA templates.

#### Metagenome Sequencing

DNA extracted from all CRV samples was quantified using the Qubit^®^ Quant-iT dsDNA High Sensitivity Kit (Invitrogen, Life Technologies, Grand Island, NY, United States) and samples were selected based on the minimum DNA concentration requirement (∼10 pg/μL) for metagenome sequencing. Only two samples from CRV1 and two samples from CRV2, with and without PMA treatment, satisfied this cutoff, resulting in 8 samples proceeding to metagenomics sequencing (2 CRV × 2 samples × 2 treatments = 8). Sample preparation for metagenome sequencing was carried out using the Illumina Nextera Kit (Illumina, San Diego, CA, United States). Each library was assessed for quality and fragment size using the Bioanalyzer 2100 (Agilent, Santa Clara, CA, United States). Adapters were added and ligated to DNA sequences in each library. All libraries were then normalized to 2 mM, pooled together and subjected to denaturation followed by dilution to 1.8 pM concentration, as per the manufacturer’s protocol. Sequencing was carried out using the HiSeq 2500 platform (Illumina, San Diego, CA, United States) resulting in 100 bp paired end reads.

### Data Processing

Adapter sequences and low-quality ends were trimmed from the 100 bp paired end reads using Trimmomatic (version 0.32) (Bolger et al., 2014) with a quality cutoff value set at minimum Phred score of 20 along the entire read length. Additionally, reads shorter than 80 bp were removed, followed by data normalization based on guidelines provided by Nayfach and Pollard (2016). Using the MEGAN 6 lowest common ancestor (LCA) algorithm (Huson et al., 2016), filtered high quality reads were binned to their respective domain and normalized for semi-quantitative comparative analysis.

Assigning taxonomy and function to the filtered CRV metagenome sequences was performed using the MEGAN 6 tool kit (Huson et al., 2016). We used the NCBI taxonomy database (Sayers et al., 2008) and NCBI-NR protein sequence database with entries from GenPept, SwissProt, PIR, PDB, and RefSeq, to assign taxonomic features to sequences using the sequence comparison tool DIAMOND (version 0.8.29). Assignments were based on the weighted LCA assignment algorithm of MEGAN 6 (Huson et al., 2007). Additionally, filtered DNA sequences were mapped against protein databases like eggnog (Powell et al., 2011), KEGG (Kanehisa and Goto, 2000), and SEED (Overbeek et al., 2005) for functional analysis.

### Comparison of CRV and ISS Metagenomes

Metagenome data from a previously reported study (Singh et al., 2018) of samples collected from the ISS were analyzed in tandem with metagenome data for samples collected from the CRV. These ISS metagenome samples were collected shortly after unberthing of CRV1 from the station or while CRV2 was berthed to the station (see Figure 1 for a timeline). Analyzing ISS and CRV metagenomes together allowed us to focus on microbial dynamics associated with the transfer of materials from CRV to the ISS. Microbial diversity analyses performed on normalized reads were designed to retain a minimum of one unique sequence to avoid the loss of low depth samples or unique sequences. Furthermore, BLAST hits of ≥20 amino acids and ≥90% nucleotide identity were used for taxonomic and functional studies. Normalized read counts for each sample at multiple taxonomic levels (e.g., domain, family, species, etc.) were exported in matrix form from MEGAN 6 and used in downstream statistical analyses.

### Statistical Analyses

To determine the differences in samples collected from the GSE and internal surfaces of both CRV1 and CRV2 and to compare them with samples collected from the ISS, several statistical analyses were performed. The non-parametric Mann–Whitney–Wilcoxon (MWW) test was used to evaluate differences in microbial diversity detected on internal surfaces of CRV and surfaces of their GSE, across CRV sampling events, and with samples collected from the ISS surfaces. The null hypothesis for the MWW test is that the distributions of microorganisms are equal between two conditions tested (e.g., internal or GSE surface), or alternatively these distributions are not equal. Parametric tests were considered for this purpose, but transformation of these data to satisfy assumptions of these tests was not practical. Samples below detection limit were assumed to have a value of 0.01 for all statistical analyses. These analyses were performed using a custom R script^1^. To measure the diversity in the microbial communities residing on the internal and GSE surfaces of CRV1 and CRV2, various statistical indices were used, including the Shannon–Weaver index (Shannon and Weaver, 1949), Simpson’s index (Simpson, 1949), and the Chao1 metric (Chao, 1984). To visualize differences in relative species abundance among CRV and ISS metagenome samples, non-metric multidimensional scaling (NMDS) using a Bray-Curtis dissimilarity matrix calculated from the normalized read counts was used. Analyses were performed using the vegan R package^2^ and custom R scripts^3^ ^,4^.

## Results

### Microbial Burden

Microbial burden assays were aimed at using both cultivation-dependent and independent methods to assess the broad diversity of microorganisms on various locations sampled from CRV1 and CRV2 (see Table 1 for a description of each sample). Since CRV3 did not show any cultivable counts, such information was not obtained.

Microbial colonies were isolated from all three locations on the GSE surfaces of CRV1 (locations 1, 2, and 7), and only one internal surface (location 4) as shown in Table 2. Using the R2A media, bacteria isolated from three GSE surfaces of CRV1 ranged between 1.3 × 10^5^ to 2.8 × 10^6^ CFU per m^2^ while 1.8 × 10^4^ CFU per m^2^ colonies were isolated from the internal location CRV1-L4, an order of magnitude lower than samples from GSE surfaces. The MWW test of colony counts on R2A media show that the three GSE surfaces of CRV1 harbor a significantly different number of cultivable bacteria than the internal locations (*W* = 12, *p*-value = 0.0436). No colonies were isolated from internal locations of CRV1 on BA media while GSE surfaces (location 2 and 7) showed 1 × 10^6^ CFU per m^2^ and location 1 showed 5.1 × 10^2^ CFU per m^2^ colonies. Fungal colonies isolated on PDA media from GSE of CRV1 ranged between 3.8 × 10^4^ to 2.9 × 10^5^ CFU per m^2^ while 2 × 10^4^ CFU per m^2^ were isolated from the internal location CRV1-L4. The MWW test of colony counts on PDA media show that the three GSE surfaces of CRV1 harbor a significantly different amount of cultivable fungi than the internal surfaces (*W* = 12, *p*-value = 0.0436).

**TABLE 2:** Total, viable, and cultivable microbiological characteristics of CRV system surface samples.

	**Cultivable bacteria**		**Cultivable fungi**	**ATP based microbial**		**Bacterial 16S rRNA**		**Fungal ITS**	
	**(CFU/m^2^)**		**(CFU/m^2^)**	**population (RLU/m^2^)**		**(copies/m^2^)**		**(copies/m^2^)**	
**Sample ID**	**R2A**	**BA**	**PDA**	**Total**	**Intracellular**	**Untreated**	**PMA-Treated**	**Untreated**	**PMA-Treated**
CRV1-L1	1.3 × 10^5^	5.1 × 10^2^	3.8 × 10^4^	4.9 × 10^5^	6.6 × 10^5^	8.6 × 10^5^	4.3 × 10^5^	7.0 × 10^5^	BDL
CRV1-L2	2.8 × 10^6^	1.0 × 10^6^	2.9 × 10^5^	9.9 × 10^6^	1.2 × 10^7^	1.0 × 10^10^	5.6 × 10^8^	1.3 × 10^6^	2.6 × 10^6^
CRV1-L3	BDL	BDL	BDL	2.8 × 10^1^	4.0 × 10^0^	3.7 × 10^5^	2.3 × 10^5^	BDL	BDL
CRV1-L4	1.8 × 10^4^	BDL	2.0 × 10^4^	1.9 × 10^5^	3.1 × 10^5^	7.2 × 10^5^	3.2 × 10^5^	7.2 × 10^4^	6.6 × 10^4^
CRV1-L5	BDL	BDL	BDL	3.1 × 10^1^	5.0 × 10^0^	5.0 × 10^5^	2.3 × 10^5^	BDL	BDL
CRV1-L6	BDL	BDL	BDL	3.1 × 10^1^	9.0 × 10^0^	3.1 × 10^5^	3.3 × 10^5^	BDL	BDL
CRV1-L7	4.0 × 10^5^	1.0 × 10^6^	1.7 × 10^5^	2.3 × 10^6^	2.6 × 10^6^	1.4 × 10^8^	6.0 × 10^7^	7.6 × 10^5^	7.7 × 10^5^
CRV1-FC	BDL	BDL	BDL	1.9 × 10^1^	6.0 × 10^0^	4.3 × 10^3^	2.7 × 10^3^	BDL	6.3 × 10^2^
CRV2-L1	3.7 × 10^4^	5.0 × 10^2^	1.5 × 10^3^	1.3 × 10^5^	9.0 × 10^4^	4.5 × 10^5^	2.8 × 10^5^	6.1 × 10^5^	BDL
CRV2-L2	BDL	BDL	BDL	6.0 × 10^1^	1.5 × 10^1^	1.9 × 10^5^	7.9 × 10^4^	2.6 × 10^5^	1.8 × 10^5^
CRV2-L3	BDL	BDL	BDL	1.8 × 10^5^	1.9 × 10^1^	2.8 × 10^5^	1.0 × 10^5^	1.3 × 10^5^	BDL
CRV2-L4	BDL	BDL	BDL	2.8 × 10^1^	3.0 × 10^0^	3.8 × 10^4^	6.1 × 10^4^	BDL	BDL
CRV2-L5	BDL	BDL	BDL	4.1 × 10^1^	2.0 × 10^0^	7.3 × 10^4^	3.6 × 10^4^	BDL	BDL
CRV2-L6	BDL	BDL	BDL	2.2 × 10^1^	8.0 × 10^0^	5.3 × 10^4^	5.4 × 10^4^	8.1 × 10^4^	BDL
CRV2-L7	3.3 × 10^3^	1.5 × 10^3^	1.5 × 10^3^	8.5 × 10^1^	1.3 × 10^1^	2.8 × 10^5^	9.5 × 10^4^	9.3 × 10^4^	BDL
CRV2-L8	9.5 × 10^2^	BDL	BDL	9.2 × 10^1^	2.4 × 10^1^	2.3 × 10^5^	6.6 × 10^4^	BDL	BDL
CRV2-FC	BDL	BDL	BDL	BDL	BDL	BDL	BDL	BDL	BDL

*BDL, below detection limit.*

CRV2 was clean as compared to CRV1 and microbial colonies were isolated from only three samples (location 1; GSE and locations 7 and 8; internal) as shown in Table 2. The microbial colonies isolated from the CRV2-L1 GSE location were 3.7 × 10^4^ and internal locations of CRV2-L7 and CRV2-L8 were 3.3 × 10^3^ and 9.5 × 10^2^ CFU per m^2^, respectively on R2A media. The MWW test of colony counts on R2A media failed to show a significant difference between bacterial counts among the internal and GSE surfaces of CRV2 (*W* = 8, *p*-value = 1.0). About 5.0 × 10^2^ (GSE location 1) and 1.5 × 10^3^ (internal location 7) CFU per m^2^ were isolated on the BA media. The MWW test of colony counts on BA media failed to show a significant difference between bacterial counts among the internal and GSE surfaces of CRV2 (*W* = 8, *p*-value = 1.0). About 1.5 × 10^3^ CFU per m^2^ of fungi were isolated from both GSE location CRV2-L1 and internal location CRV2-L7 on the PDA media. The MWW test of colony counts on PDA media failed to show a significant difference between fungal counts between the internal and GSE surfaces of CRV2 (*W* = 8.5, *p*-value = 0.8437). The microbial colony counts for the CRV2 were lower by almost an order of magnitude as compared to CRV1. However, the MWW test failed to show a significant difference in colony counts between CRV1 and CRV2 on R2A media (*W* = 38.5, *p*-value = 0.2094), BA media (*W* = 35, *p*-value = 0.3698), or PDA media (*W* = 41, *p*-value = 0.1023). Strikingly, no samples from GSE or internal surfaces of the CRV3 vehicle showed any trace of a microbial population.

Identification of cultured microbes revealed the presence of four bacterial phyla (*Actinobacteria*, *Proteobacteria*, *Firmicutes*, and *Bacteroidetes;* Supplementary Table S1) and two fungal phyla (*Ascomycota* and *Basidiomycota*; Supplementary Table S1). Almost all the bacterial isolates, besides *Pseudarthrobacter*, *Compostimonas*, and *Mycobacterium*, have also been detected by the metagenome derived diversity analysis, at least at the genus level, whereas half of the fungal isolates (*Bullera/Papiliotrema*, *Cryptococcus/Hannaella/Naganishia*, *Cladosporium*, *Dioszegia*, *Rhodotorula*, *Periconia*, *Phialemoniopsis*, and *Phlebia*) were not detected.

Cultivation-independent methods used to estimate the microbial burden included ATP and qPCR assays. The intracellular ATP assay quantified the content of live cells, and total ATP quantified both dead and live cells to provide an estimate of the viable microbial population in a given sample. In the case of CRV1, the total ATP content for the GSE surfaces ranged between 10^5^ to 10^6^ RLUs per m^2^, while all internal surface locations had ATP content lower by four orders of magnitude, except for the CRV1-L4 location which had 1.9 × 10^5^ RLUs per m^2^ (Table 2 and Supplementary Figure S1). The intracellular ATP content for all GSE surfaces and one internal surface (CRV1-L4) were similar to their respective total ATP content, thus indicating that almost 100% of the microorganisms were viable at these locations. However, the intracellular ATP content was considerably lower for other internal locations of CRV1 and indicated that only 15–30% of the total microorganisms were viable. Incidentally, cultivable populations were isolated only from the CRV1-L4 sample. In the case of the CRV2, all GSE surfaces showed total ATP content of 10^5^ RLUs per m^2^, except for CRV2-L2 which had only 10^1^ RLUs per m^2^ (Table 2 and Supplementary Figure S1). The total ATP RLU values for all internal locations of CRV2 were similar to CRV1 with only 10^1^ RLUs per m^2^. The MWW test failed to show a significant difference in total or intracellular ATP content between CRV1 and CRV2 (*W* = 45.5, *p*-value = 0.3859 or *W* = 49, *p*-value = 0.2359). The intracellular ATP content for all internal locations of CRV2 was lower than the respective total ATP content thus indicating that 5–36% of the total microorganisms were viable.

The qPCR-based DNA quantification using the 16S rRNA gene and the ITS region provided an estimate of the bacterial and fungal burden of the CRV1 and CRV2. In the case of CRV1, the PMA treated samples (viable microbes) from the GSE sample locations had very high bacterial density for locations 2 and 7, ranging between 10^7^–10^8^ 16S rRNA gene copies per m^2^ (Table 2 and Supplementary Figure S2A). However, location 1 (GSE) and all internal locations of CRV1 showed 10^5^ viable bacterial population per m^2^. In general, the PMA treated CRV1 samples showed 16S rRNA gene copy numbers lower by one to two orders of magnitude for the intact cells from GSE locations samples, while there was an only minute difference in the intact cell population for all internal CRV surface samples. The percentage of the intact cells for both internal and GSE locations of CRV1 ranged between 40 and 64%, except for the location 2 and 6, which had 5 and 100% of the intact cells, respectively.

Similarly, the fungal density estimated using quantification of the ITS region showed that the ITS gene copies in PMA untreated samples in CRV1 samples were less by three to four orders of magnitude as compared to their bacterial counterpart. The ITS copies for the PMA untreated samples ranged between 10^5^ and 10^6^ for the GSE locations and 10^4^ ITS copies per m^2^ for internal locations (Table 2 and Supplementary Figure S2B). The PMA treated samples for both internal and GSE CRV1 locations showed similar ITS copy numbers to the PMA untreated samples, thus making the percentage of intact fungal cells 90–100% for CRV1-L4 and CRV1-L7. However, the GSE location CRV1-L2 showed only 20% of the intact fungal population.

In the case of CRV2, the 16S rRNA gene copy numbers of PMA untreated samples for the GSE and internal locations were 10^5^ and 10^4^ copies per m^2^, respectively. The percentage of the intact bacterial cell numbers determined from the PMA treated samples for both GSE and internal locations were in the range of 36–62% and 34–100%, respectively. The fungal density for the CRV2 samples for both GSE and internal locations for the PMA untreated samples was in the range of 10^5^ and 10^4^, respectively. Additionally, these fungal cell numbers were higher by an order of magnitude as compared to their bacterial counterpart.

The MWW test of 16S rRNA gene copy numbers of PMA treated samples showed a significant difference in bacterial abundance between CRV1 and CRV2 (*W* = 62, *p*-value = 0.01408). The MWW test of ITS copy numbers of PMA treated samples, however, failed to demonstrated a significant difference in fungal abundance between CRV1 and CRV2 (*W* = 50, *p*-value = 0.107). When comparing 16S rRNA or ITS copy numbers from GSE and internal samples, the MWW test failed to find any significant difference (*M* = 43, *p*-value = 0.0675 or *M* = 39, *p*-value = 0.08197, respectively).

### Metagenome Derived Microbial Diversity

Samples were collected from three CRV vehicles (25 samples in total; Table 1) and each wipe was either treated with PMA or left untreated, resulting in an analysis of 50 samples, including 6 controls. Among the 50 samples subjected for shotgun library preparation, only 8 samples (4 non-PMA and corresponding 4 PMA treated) yielded shotgun metagenome libraries (none from the controls) and all other 42 samples were below the detection limit (0.01 ng DNA/μL). Subsequently, these low DNA concentration samples (42 samples) did not produce any shotgun metagenome sequences. High quality metagenome reads were obtained for four samples; one each from GSE (CRV1-L2; CRV2-L1) and internal (CRV1-L4; CRV2-L7) locations. Interestingly, the microbial burden (both cultivable and qPCR assays) of these four sites were high when compared to other locations (Table 2).

At the domain level, bacteria dominated the microbial diversity detected in the metagenomes sampled from internal and GSE surfaces of CRV1 and CRV2. In the case of CRV1, bacteria at both internal (CRV1-L4) and GSE (CRV1-L2) locations constituted 99 and 88% of the total reads from PMA treated samples, respectively (Supplementary Figure S3). PMA treated samples from CRV2 were very similar to CRV1 samples when compared at the domain level, both GSE (CRV2-L1) and internal (CRV2-L7) locations were dominated by bacteria (90 and 94%, respectively). Most of the remaining reads belonged to Eukaryota, constituting <1–12% of the total reads from CRV1 and CRV2 internal and GSE locations. The viral and archaeal reads constituted a very minute fraction of the total reads in PMA treated samples from internal and GSE locations of CRV1 and CRV2.

At the phylum level (Supplementary Figure S4), the PMA treated samples showed significant differences between internal and GSE locations of both CRV1 and CRV2. The CRV1 GSE was dominated by Proteobacteria (95%) while the internal locations majorly constituted of Proteobacteria (32%), Firmicutes (29%), Actinobacteria (21%), followed by Ascomycota (8%). The CRV2 GSE also showed dominance of Proteobacteria reads (53%) followed by Actinobacteria (11%), Firmicutes (10%), and Ascomycota (7%). The CRV2 internal location had a different composition as compared to the GSE at the phylum level, which was dominated by Firmicutes (40%), closely followed by Proteobacteria (32%), and Actinobacteria (16%) reads. Also, the internal locations of CRV1 and CRV2 showed relatively similar abundance at the phylum level except for Actinobacteria, Firmicutes, and Ascomycota, as mentioned earlier.

At the class level (Supplementary Figure S5), the PMA treated GSE samples of CRV1 were dominated by Betaproteobacteria (73%) followed by Alphaproteobacteria (11%), while the internal location was dominated in descending order by Bacilli (28%), Actinobacteria (21%), Gammaproteobacteria (18%), Alphaproteobacteria (8%), Dothideomycetes (6%), and Betaproteobacteria (4%). In the case of CRV2 PMA treated samples, the GSE showed a high abundance of Gammaproteobacteria (21%), followed by Betaproteobacteria (17%), Actinobacteria (11%), Alphaproteobacteria (10%), and Bacilli (10%). On the other hand, Bacilli (39%) dominated the internal location of CRV2 PMA treated samples, followed by Betaproteobacteria (18%), Actinobacteria (15%), and Alphaproteobacteria (8%). Additionally, significant differences were observed at the class level for the internal locations of both CRV1 and CRV2.

At the order level (Supplementary Figure S6), Burkholderiales constituted more than 71% of the reads for the PMA treated samples from the GSE of CRV1, which was distantly followed by Caulobacterales (7%), and Sphingomonadales (3%). The internal location of CRV1 presented a completely different picture, with only 4% of the reads belonging to Burkholderiales, while the most dominant order was Bacillales (27%), followed by Pseudomonadales (16%), and Propionibacteriales (10%). Furthermore, reads for Caulobacterales and Sphingomonadales were detected at very low levels (<1%) from the internal location of CRV1. The PMA treated samples for CRV2 internal and GSE surfaces showed a similar trend with respect to the order level. Burkholderiales, Bacillales, Pseudomonadales, and Propionibacteriales were the dominant orders detected on both internal and GSE surfaces. However, the only difference between GSE and internal CRV2 samples was that the reads belonging to Bacillales were dominant at the internal location (36%) and Pseudomonadales was dominant for GSE (18%).

At the family level (Supplementary Figure S7), large differences were observed on the internal and GSE surfaces of CRV1 for the PMA treated samples. Reads for *Oxalobacteraceae* (33%), *Comamonadaceae* (22%), and *Caulobacteraceae* (6%) dominated the GSE surface of CRV1, followed by *Sphingomonadaceae* (3%) and *Burkholderiaceae* (1%). The internal location for CRV1 showed the complete opposite trend with only 4% of the reads belonging to *Oxalobacteraceae* family, while *Comamonadaceae*, *Caulobacteraceae*, *Sphingomonadaceae*, and *Burkholderiaceae* were detected at very low levels (<1%). Instead, the CRV1 internal location was dominated by *Pseudomonadaceae* (15%), *Bacillaceae* (10%), *Propionibacteriaceae* (10%), and *Aureobasidiaceae* (5%) families. The CRV2 PMA treated samples for both internal and GSE surfaces were relatively similar at the family level, mainly belonging to *Oxalobacteraceae*, *Bacillaceae, Pseudomonadaceae*, and *Propionibacteriaceae*. However, *Bacillaceae* was dominant on the internal surface (13%) while *Oxalobacteraceae* and *Pseudomonadaceae* were dominant on the GSE surface (12 and 11%, respectively) for the PMA treated CRV2 samples. Additionally, reads belonging to a fungal family *Pleosporaceae* was present in abundance at only the GSE surface (3%) of CRV2.

At the genera level (Supplementary Figure S8), as observed previously for other taxon levels, the PMA treated samples of CRV1 showed large differences between the GSE and internal surfaces. Human commensal *Massilia* (25%), *Acidovorax* (15%), and *Caulobacter* (6%) were the most dominant genus detected at the GSE surface of CRV1. However, reads for the genus *Acidovorax*, and *Caulobacter* were only detected at low levels (<1%) from the internal location of CRV1 and *Massilia* constituted only 3% of the total reads. The internal location of CRV1 was instead dominated by the bacterial genera *Pseudomonas* (15%) and *Bacillus* (9%), and the fungal genus *Aureobasidium* (5%). On the other hand, both the GSE and internal surfaces of CRV2 presented a relatively similar pattern of genera, including *Massilia*, *Acidovorax*, *Pseudomonas*, *Bacillus*, and *Caulobacter*. Abundances of these genera were different between the GSE and internal surfaces, with *Pseudomonas* (11%) and *Massilia* (8%) being dominant on the GSE surface, while the internal surface showed an abundance of *Bacillus* (12%), *Massilia* (7%), and *Acidovorax* (2%).

**FIGURE 2:**
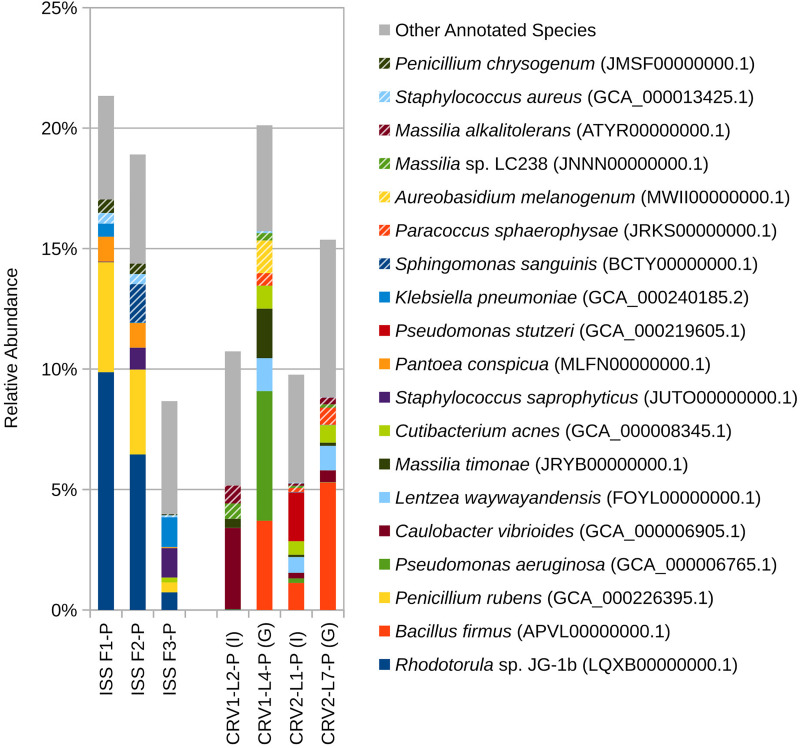
Relative abundance of species based on shotgun metagenomic reads generated from PMA treated samples collected from three sampling events on the ISS and the internal (I) and ground support equipment (G) surfaces of CRV1 and CRV2.

At the species level (Figure 2), PMA treated samples for the internal and GSE surfaces of both CRV1 and CRV2 presented a clear distinction in terms of species abundance. *Caulobacter vibrioides* is the only microbial species detected in abundance (3%) on the GSE surface of CRV1. The internal location, however, showed a completely different trend with *Pseudomonas aeruginosa* being the most abundant member, comprising 5% of total microbial abundance, followed by *Bacillus firmus* (4%), *Massilia timonae* (2%), *Lentzea waywayandensis* (1%), and the fungus *Aureobasidium melanogenum* (1%). The CRV2 PMA treated samples for the internal location showed the presence of *Bacillus firmus* (5%), *Cutibacterium acnes* (1%), and *Lentzea waywayandensis* (1%). In the sample collected from the GSE surface of CRV2, *Pseudomonas stutzeri* (2%), *Bacillus firmus* (1%), *Cutibacterium acnes* (1%), and *Lentzea waywayandensis* (1%) were present at the outside location of CRV2 samples.

### Statistical Significance

To determine the differences at various taxon levels between the PMA treated and untreated samples from the GSE and internal surfaces of both CRV1 and CRV2, several statistical analyses were performed. Based on the Mann–Whitney–Wilcoxon statistics (Supplementary Table S2), no significant differences (*p* > 0.05) were observed at the domain and phylum levels between the GSE and internal surfaces of PMA treated samples of CRV1. The class, family, order, genus, and species levels showed significant differences (*p* < 0.05) between the GSE and internal surfaces of PMA treated samples for CRV1. In the case of CRV2, no significant differences (*p* > 0.05) were observed at the domain, phylum, and class levels between the GSE and internal surfaces of PMA treated samples although, family, order, genus, and species levels were significantly different (*p* < 0.05). Similar results were also seen for the PMA untreated samples, except for the CRV2 samples where phylum and class also showed significant difference (*p* = 0.0077 and *p* = 0.0004, respectively) between the internal and GSE surfaces. On comparing the PMA treated samples at internal locations between the CRV1 and CRV2, the Mann–Whitney–Wilcoxon statistics failed to detect any differences at the domain, class, and species level (*p* > 0.05), with phylum, order, family, and genera level being significantly different (*p* < 0.05). On the other hand, the untreated samples were significantly different (*p* < 0.05) between CRV1 and CRV2 at the internal locations except for the domain and species level (*p* = 0.1353 and *p* = 0.0918, respectively). The PMA treated as well as untreated samples for the GSE surfaces were significantly different (*p* < 0.05) for all taxon levels at the CRV1 and CRV2, except for the domain and phylum level (*p* = 0.0647 and *p* = 0.2069, respectively).

**FIGURE 3:**
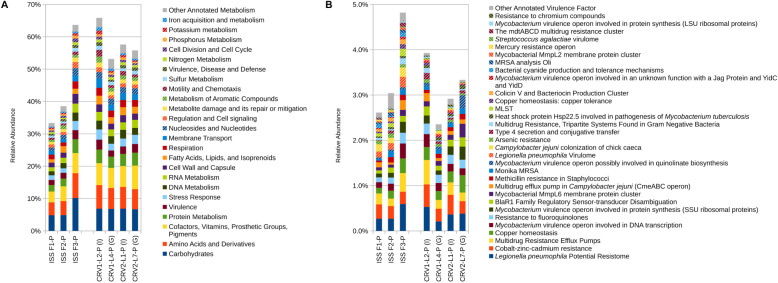
Relative abundance of metagenomic reads associated with **(A)** microbial metabolism and **(B)** virulence. Metagenomic reads were generated from PMA treated samples collected from three sampling events on the ISS and the internal (I) and ground support equipment (G) surfaces of CRV1 and CRV2.

Subsequently, to measure the diversity in the microbial communities residing on the internal and GSE surfaces of CRV1 and CRV2, various statistical indices were used (Figures 3A–C). The Shannon–Weaver index *H* values for the PMA treated samples were consistently lower than the PMA untreated samples on the internal and GSE surfaces of both CRV1 and CRV2. The *H* values can thus be interpreted as an increase in the species richness and evenness of the untreated samples as compared to the PMA treated samples. In the case of CRV1, the internal surface showed higher *H* values compared to the GSE surface (both PMA treated and untreated) thus indicating high species richness at the internal location. However, a completely opposite trend was observed for CRV2 where the GSE surface showed higher species richness and evenness as compared to the internal surface. A similar comparison made between the *H* values of the internal and GSE surfaces of CRV1 and CRV2 PMA treated samples showed that the CRV2 had higher species richness as compared to CRV1. Diversity analysis carried out using the Simpson index further confirmed the Shannon–Weaver index findings. Another diversity index, the c index, showed that the species diversity increased for the PMA untreated samples as compared to that of the PMA treated samples on the internal and GSE surfaces of both CRV1 and CRV2. Similarly, in the case of CRV2, the untreated samples showed more species diversity compared to PMA treated on both internal and GSE surfaces. Additionally, the comparison of PMA treated samples at internal locations of CRV2 and CRV1 showed that the CRV2 had more species diversity as compared to the CRV1.

### Functional Genes

Reads associated with metabolic functions like amino acid derivatives, carbohydrates, cofactors and vitamin metabolism, and protein metabolism were most abundant and relatively equally distributed for all the PMA treated samples on the internal and GSE surfaces of CRV1 and CRV2 as shown in the (Figure 3A). However, genes corresponding to other important functions like membrane transport, DNA and RNA metabolism, nucleotide metabolism, cell wall synthesis, and metabolism of aromatic compounds showed major differences between the internal and GSE surfaces of CRV1 and CRV2, and were statistically significant (Supplementary Table S2, *p* < 0.05). Furthermore, the MWW analysis (Supplementary Table S2) showed significant differences (*p* < 0.05) between the internal locations of CRV1 and CRV2 as well as the GSE surfaces of CRV1 and CRV2.

### Virulence Genes

The metagenome reads for internal and GSE surfaces of both CRV1 and CRV2 were also subjected to virulence gene analyses using the virulence factors from the SEED database as reference. A major fraction of the virulence genes belonged to multiple drug resistance (MDR) efflux pump, *Legionella pneumophila* potential resistome, and Cobalt-Zinc-Cadmium resistance genes as shown in Figure 3B. Furthermore, these genes showed relatively equal distribution on both internal and GSE surfaces of CRV1 and CRV2. The relative abundance of the reads associated with virulence factors were vastly different on the internal location as compared to the GSE surfaces for both CRV1 and CRV2, and were statistically significant (Supplementary Table S2, *p* < 0.05). The MWW method (Supplementary Table S2) additionally showed statistically significant differences (*p* < 0.05) between the internal locations of CRV1 and CRV2 as well as the GSE surfaces of CRV1 and CRV2 (*p* = 0.05).

### Antimicrobial Resistance

**FIGURE 4:**
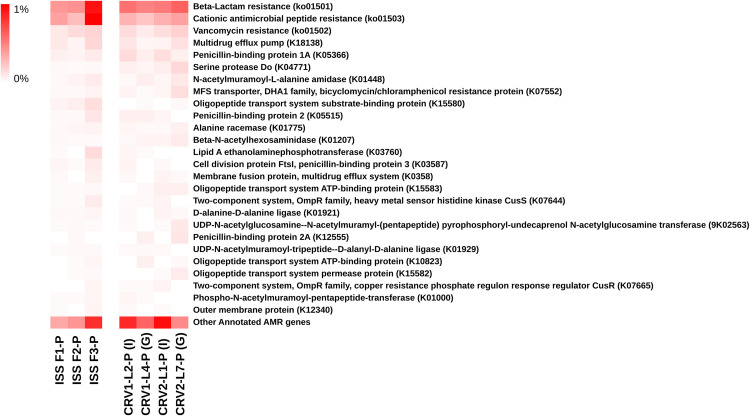
Distribution of the antibiotic resistance genes detected in metagenomes generated from PMA treated samples collected from the internal (I) and ground support equipment (G) surfaces of CRV1 and CRV2 and three sampling events on the ISS.

Metagenomic reads of PMA treated samples of internal and GSE surfaces of CRV1 and CRV2 were screened for antimicrobial resistance (AMR) genes. Based on the percent abundance, the AMR genes were sorted into different categories as shown in (Figure 4). Beta-lactam resistance, Cationic antimicrobial peptide (CAMP) resistance, and Vancomycin resistance together comprised a major fraction of the reads belonging to AMR genes and were also relatively equally distributed across internal and GSE surfaces of both CRV1 and CRV2. The remaining half of the AMR genes were mainly comprised of penicillin binding protein, multidrug efflux pump, and serine protease genes. Importantly, except for the MDR efflux pump there was no overlap between the virulence and AMR genes. Therefore, an independent analysis was performed for AMR and virulence genes.

### Comparative Analyses of ISS and CRV Microbial Diversity

Two bacterial species and one fungal species that were isolated during this study of CRV surfaces (Supplementary Table S1) were also isolated from the ISS surfaces by Checinska Sielaff et al. (2019). Strains of the common skin commensal bacterium and opportunistic pathogen, *Staphylococcus epidermidis*, were isolated from CRV (*N* = 3) and ISS (*N* = 6) surfaces. Strains of *Micrococcus yunnanensis*, a bacterium previously detected within plant roots (Zhao et al., 2009), were isolated from CRV (*N* = 5) and ISS (*N* = 1) surfaces. Strains of *Rhodotorula mucilaginosa*, a common environmental saprophytic yeast and opportunistic pathogen, were also isolated from CRV (*N* = 1) and ISS (*N* = 33) surfaces. The other 45 bacterial species and 19 fungal species isolated from CRV surfaces were not isolated from ISS surfaces.

**FIGURE 5:**
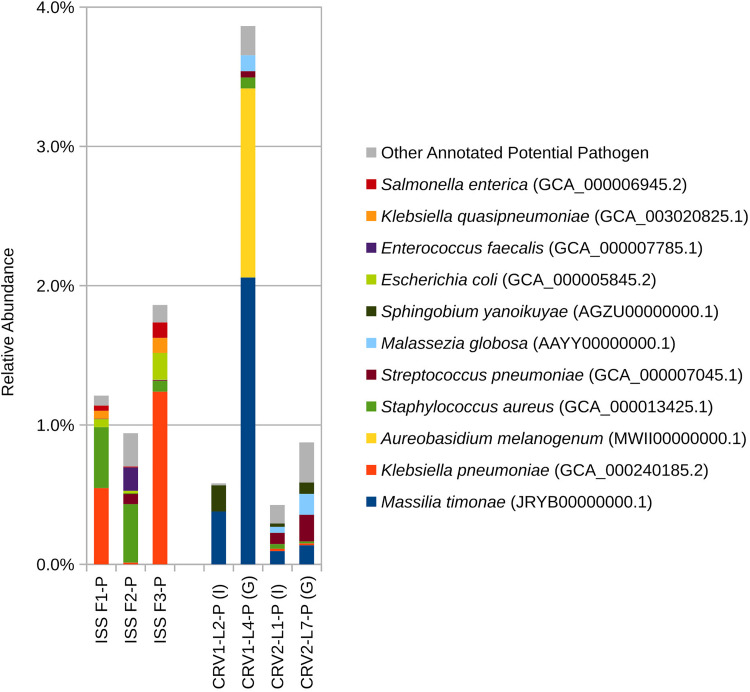
Relative abundance of potential opportunistic pathogenic microorganisms detected in metagenomes generated from PMA treated samples collected from the internal (I) and ground support equipment (G) surfaces of CRV1 and CRV2 and three sampling events on the ISS.

Eight bacterial species and one fungal species were detected in both ISS and CRV microbiome samples, including human commensal and environmental microbes. *Cutibacterium acnes*, a common skin commensal, could be detected at low levels in ISS samples (<2%) and CRV samples (<1%). *Klebsiella pneumoniae*, a common nosocomial pathogen, could be detected at varying levels in ISS samples (0–7%) but at very low levels in CRV samples (≤0.1%). Similarly, *Staphylococcus aureus*, a common commensal bacterium also known to be an opportunistic pathogen, was detected at varying levels in ISS samples (0–3%) but at very low levels in CRV samples (≤0.1%, Figure 5). *Streptococcus pneumoniae*, another common commensal bacterium that is also an opportunistic pathogen, was detected at low abundance in ISS and CRV microbiome samples (<1% and <0.2%, respectively). *Methylobacterium* sp. ME121, an environmental bacterium, could be detected at low levels in ISS samples (<1%) and very low levels in CRV samples (<0.1%). *Sphingobium yanoikuyae*, an environmental bacterium known to degrade polycyclic aromatic hydrocarbons, was detected at very low abundance in ISS samples (<0.1%) and low abundance in CRV samples (<0.2%). Environmental bacteria *Sphingomonas* sp. Ag1 and *Sphingomonas* sp. OV641 were also detected at very low abundance in ISS (<0.2%) and CRV (<0.3%) microbiome samples. *Rhodotorula* sp. JG-1b, a psychrophilic fungus, was detected at varying levels in the ISS microbiome samples (0–40%), but at very-low levels in CRV samples (<0.01%).

**FIGURE 6:**
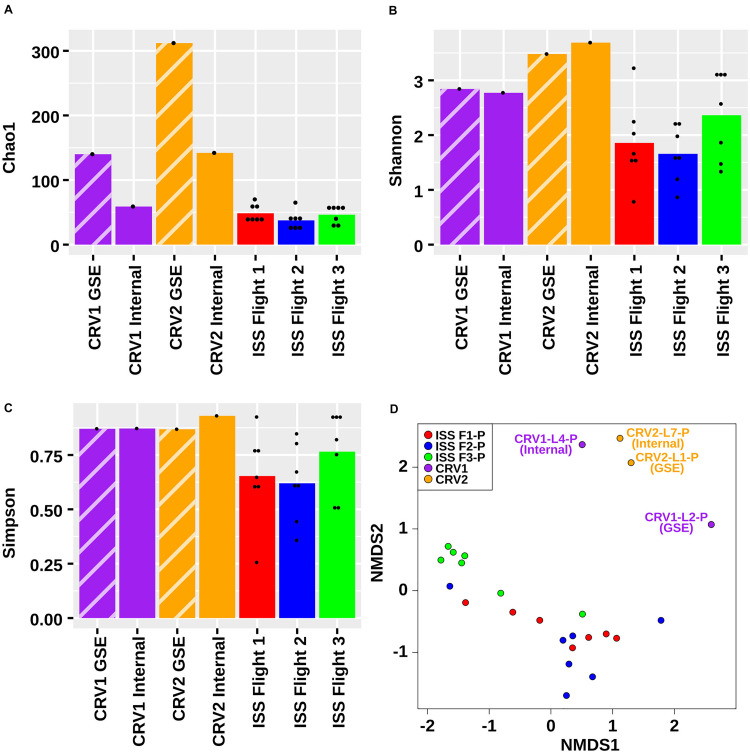
Chao1 **(A)**, Shannon **(B)**, Simpson **(C)**, and NMDS **(D)** plots representing the differences in the microbial diversity detected from metagenomes generated from PMA treated samples collected from the internal (I) and ground support equipment (GSE) surfaces of CRV1, CRV2 and the locations from three sampling events of the ISS.

Although there was some overlap in species detected among ISS and CRV samples, the vast number of species detected did not overlap (309 and 111 species, respectively). Microbial species indices as calculated by Chao1 (Figure 6A), Shannon (Figure 6B), and Simpson (Figure 6C) also supported that microbial diversity was different between CRV and ISS environmental surface samples. When the overall abundance of species detected in each sample is visualized in an NMDS ordination (Figure 6D), this relationship becomes apparent. The microbiomes sampled in the ISS (red, blue, and green glyphs) cluster separately in the ordination from the microbiomes sampled in CRV1 (purple glyphs) and CRV2 (orange glyphs). The MWW test showed a significant difference in species abundance between CRV and ISS samples (*W* = 246120, *p*-value = 0.000162). Significant differences between ISS and CRV microbiomes could also be detected in genes associated with virulence (*W* = 4308.5, *p*-value = 1.443e-07) and AMR (*W* = 30626, *p*-value < 2.2e-16). However, no significant difference could be detected in genes associated with metabolism between the ISS and CRV (*W* = 1210, *p*-value = 0.4271).

## Discussion

The current study aims at understanding the source of contamination of the ISS by characterizing the microbial diversity of the CRV just before launching. The ISS’s robotic Canadarm appendage captures CRV carrying payloads for the ISS crew, after the CRV reaches the orbit of the ISS. As a result, in the current study, we compare the microbial diversity of the CRV with that of the previously reported ISS microbiome (Singh et al., 2018; Checinska Sielaff et al., 2019).

Most of the CRV cultivable isolates were already reported from samples collected from SAF cleanroom environments (Moissl-Eichinger et al., 2013), with the exception of *Compostimonas*. Most of these CRV isolates have been identified as species normally associated with soils, whereas cultivable microorganisms from the ISS were primarily associated with humans (*Staphylococcaceae* and *Enterobacteriaceae*) with the exception of *Bacillus* that was common in both CRV and ISS (Checinska Sielaff et al., 2019). When culturable fungi were compared, most of the CRV fungal members were also present in the ISS (Checinska Sielaff et al., 2019). However, strain specific similarities of these fungal isolates will need to be carried out via a sophisticated strain-tracking molecular method in the future. The CRV isolates that pose a potential health risk for astronauts that may be immunocompromised are *Staphylococcus pasteuri* (e.g., bacteraemia) (Savini et al., 2009), *Exophiala xenobiotica* (e.g., phaeohyphomycosis) (Aoyama et al., 2009), and *Phialemonium dimorphosporum* (e.g., fungemia) (Guarro et al., 1999). The cultivable strains also included members of the genera *Bacillus* and *Paenibacillus*, which are often encountered in SAF environments and can withstand harsh conditions (e.g., desiccation, low nutrients, and radiation) because of their spore formation ability (Vaishampayan et al., 2012). In addition, *Bacillus* and *Sphingomonas* are also known to form biofilms, which may increase various resistance capabilities of these microorganisms, and therefore also lead to an increased contamination risk (Checinska Sielaff et al., 2019).

The metagenome sequence analysis of the GSE and internal surfaces of CRV1 and CRV2 grouped separately when compared with the eight locations from the ISS environment sampled during three flights from a previously reported study by Singh et al. (2018) (Figure 6). Furthermore, the microbiome profiles of the internal CRV were different from GSE of the CRV. The PMA treated samples collected from the GSE surface of the CRV1 were dominated by reads belonging to genera *Acidovorax*, *Caulobacter*, and *Massilia* (reclassified as *Naxibacter*), while the CRV2 GSE surface showed *Pseudomonas* and *Bacillus*. These bacteria are known to survive extreme oligotrophic conditions for extended time periods (Wilhelm, 2018; Gray et al., 2019). Additionally, the members of the aforementioned genera are known to tolerate alkaline (Yumoto et al., 2001; Xu et al., 2005) and oxidative stress conditions (Lan et al., 2010), and survive high levels of ultraviolet radiation (Vaishampayan et al., 2012). Similarly, routine maintenance and cleaning procedures could presumably have removed most of the bacterial taxa associated with the SAF cleanrooms, however, spore-forming bacteria and members of Actinobacteria and *Massilia* persist in these environments (Vaishampayan et al., 2013; Mahnert et al., 2015).

The PMA treated samples collected from the internal locations of CRV1 and CRV2 were dominated by *Bacillaceae*, *Pseudomonadaceae*, *Propionibacteriaceae*, and *Oxalobacteraceae*, and were different from the ISS microbiome which was dominated by *Enterobacteriaceae*, *Methylobacteriaceae*, *Staphylococcaceae*, *Aspergillaceae*, and *Sporidiobolaceae*. Furthermore, the CRV microbiomes showed similarities with the SAF microbiome (Vaishampayan et al., 2013; Mahnert et al., 2015). The species level comparison between the ISS and CRV microbiomes (cut off value of minimum 10 reads) showed that only nine microorganisms were common between the two ecosystems. Except for *Methylobacterium*, all other microbes were common skin commensals (e.g., *Cutibacterium acnes*, *Enterobacter cloacae*, *Malassezia globosa*, *Propionibacterium humerusii*, *Staphylococcus aureus*, and *Streptococcus pneumoniae*). Additionally, the CRV also showed the presence of the members of genera belonging to different ecosystems (indoor to aquatic environments), like *Pseudomonas*, *Mycobacterium*, *Massilia*, *Leifsonia*, *Corynebacterium*, *Caulobacter*, *Bacillus*, *Acidovorax*, and *Aureobasidium*. It is also interesting to note that some of the microorganisms that were cultured were not detected in a shotgun metagenome sequencing approach. This might be due to the fact that ITS-based identification of fungi was not always ideal in determining the species accurately. Further research on the WGS of cultured microorganisms would reveal the authentic identification.

In general, most of the species observed in the CRV microbiome could not be observed in the ISS microbiome, potentially because the ISS has a consistent human presence. On the other hand, although the CRV assembly facility has constant human traffic, the cleanrooms are continuously undergoing cleaning and maintenance procedures. It is also important to note that cleaning aboard the ISS occurs sporadically and mainly during the weekend. Whereas daily stringent cleaning regiment occurs inside the CRV assembly facility to maintain a low biomass. Additionally, chemical used to clean inside the CRV are based on wipes with 3% hydrogen peroxide, whereas aboard the ISS the crew use wipes with 0.1% dimethyl ammonium chloride derivatives. Difference seen between the CRV sampled could be also due to differences in the time of the year the samples were collected. Furthermore, it is important to note that cleaning within the CRV1 was done 24 h prior to sampling. Whereas for CRV2 and CRV3, it was done the day of the sampling event and for the latter just prior to our arrival. Another potential reason for the absence of CRV microbiome in the ISS microbiome would be the limitations of the sequencing procedures. Being dominated by the human microbiome, sequences of rare or extremely low abundance microorganisms may not be detected in the ISS microbiome. Therefore, even though a certain low number of microorganisms are carried via CRV to the ISS, most of them will be outcompeted by the existing ISS microbiome, potentially rendering them undetectable in both traditional microbiology assays and state-of-the-art metagenome sequencing. The limitations of this study are related to the extremely low biomass of the CRV samples collected. When examining metagenome positive samples, it is apparent that they harbor more cultivable bacteria and fungi which might require appropriate cultivation conditions.

In conclusion, this is the first report that demonstrates, using both traditional microbiological methods and molecular techniques, that there is a very little risk of transferring microorganisms to the ISS owing to CRV transport. Significant differences were shown between the ISS and the CRV microbiomes using statistical methods. The cleaning protocols for CRV surfaces appear to be highly effective, and continued observance of these protocols is recommended to limit forward contamination of the ISS.

## Data Availability Statement

The datasets generated for this study can be found in online repositories. The names of the repository/repositories and accession number(s) can be found below: https://www.ncbi.nlm.nih.gov/, PRJNA448453 and https://genelab.nasa.gov/, GLDS-286.

## Author Contributions

SM, JW, and KV wrote the manuscript. JW generated all figures in the manuscript. NS processed the shotgun metagenome sequencing data. JW performed all statistical analyses. KV and AS were involved in study design, helped interpret and write the manuscript, processed the samples, and performed culture and qPCR analysis. MM, SD, and CM-E prepared Sanger sequencing data and performed culturable diversity analyses. JW performed a comparative analysis of the ISS surface and CRV microbiomes. FK was instrumental in science payload, metadata generation, and coordinated with CRV institution in sample collection. KV was involved in early organization, study design and planning of the research project, and providing direct feedback to all authors throughout the project and during write-up of the manuscript. All authors read and approved the final manuscript.

## Disclaimer

This manuscript was prepared as an account of work sponsored by NASA, an agency of the United States Government. The United States Government, NASA, California Institute of Technology, Jet Propulsion Laboratory, and their employees make no warranty, expressed or implied, or assume any liability or responsibility for the accuracy, completeness, or usefulness of information, apparatus, product, or process disclosed in this manuscript, or represents that its use would not infringe upon privately held rights. The use of, and references to any commercial product, process, or service does not necessarily constitute or imply endorsement, recommendation, or favoring by the United States Government, NASA, California Institute of Technology, or Jet Propulsion Laboratory. Views and opinions presented herein by the authors of this manuscript do not necessarily reflect those of the United States Government, NASA, California Institute of Technology, or Jet Propulsion Laboratory, and shall not be used for advertisements or product endorsements.

## Conflict of Interest

The authors declare that the research was conducted in the absence of any commercial or financial relationships that could be construed as a potential conflict of interest. The reviewer AR declared a shared affiliation with several of the authors SM, JW, AS, NS, and KV.

## Footnotes

1 https://github.com/sandain/R/blob/master/mw.R

2 https://cran.r-project.org/web/packages/vegan/

3 https://github.com/sandain/R/blob/master/diversity.R

4 https://github.com/sandain/R/blob/master/mds.R

## Supplementary Material

Supplementary Figure 1 Total and Intracellular ATP measured in field control (FC) and surface samples collected from CRV1 and CRV2.

Supplementary Figure 2 Number of copies of **(A)** bacterial 16S and **(B)** fungal ITS detected via qPCR in CRV1 and CRV2 samples with and without PMA treatment. Field controls (FC), no template controls (NTC), extraction controls (Maxwell controls; MC), and positive controls (PC) are included for comparison.

Supplementary Figure 3 Relative abundance of domain based on shotgun metagenomic reads generated from PMA treated samples collected from three sampling events on the ISS and the internal (I) and ground support equipment (G) surfaces of CRV1 and CRV2.

Supplementary Figure 4 Relative abundance of phyla based on shotgun metagenomic reads generated from PMA treated samples collected from three sampling events on the ISS and the internal (I) and ground support equipment (G) surfaces of CRV1 and CRV2.

Supplementary Figure 5 Relative abundance of classes based on shotgun metagenomic reads generated from PMA treated samples collected from three sampling events on the ISS and the internal (I) and ground support equipment (G) surfaces of CRV1 and CRV2.

Supplementary Figure 6 Relative abundance of orders based on shotgun metagenomic reads generated from PMA treated samples collected from three sampling events on the ISS and the internal (I) and ground support equipment (G) surfaces of CRV1 and CRV2.

Supplementary Figure 7 Relative abundance of families based on shotgun metagenomic reads generated from PMA treated samples collected from three sampling events on the ISS and the internal (I) ground support equipment (G) surfaces of CRV1 and CRV2.

Supplementary Figure 8 Relative abundance of genera based on shotgun metagenomic reads generated from PMA treated samples collected from three sampling events on the ISS and the internal (I) and ground support equipment (G) surfaces of CRV1 and CRV2.
