# Supplementary figures and images for: Assessing the Risk of Transfer of Microorganisms at the International Space Station Due to Cargo Delivery by Commercial Resupply Vehicles
Source: Front Microbiol. 2020 Nov 6;11:566412. doi: 10.3389/fmicb.2020.566412 (PMC7677455; doi:10.3389/fmicb.2020.566412)

S1

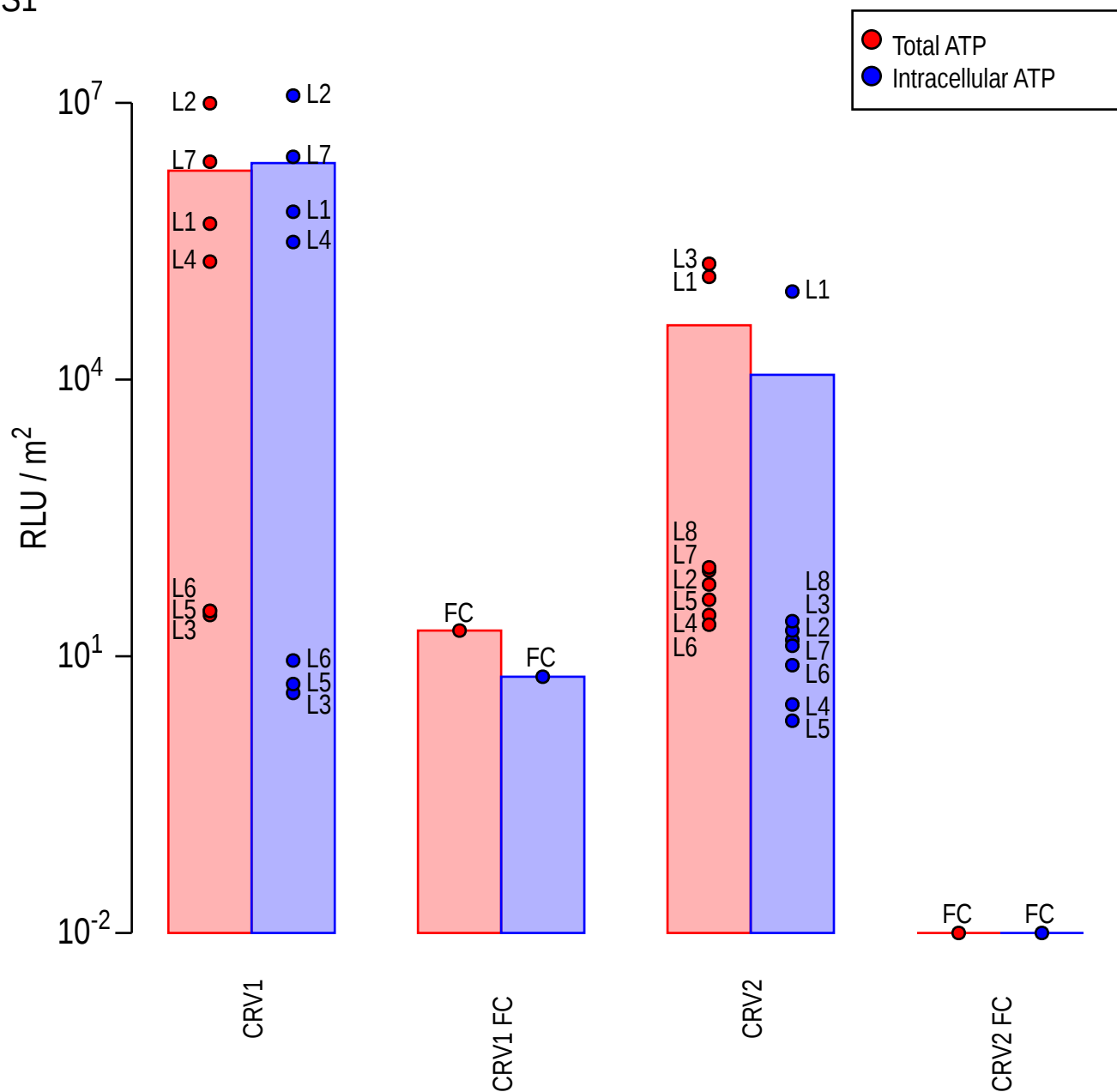

S2

A

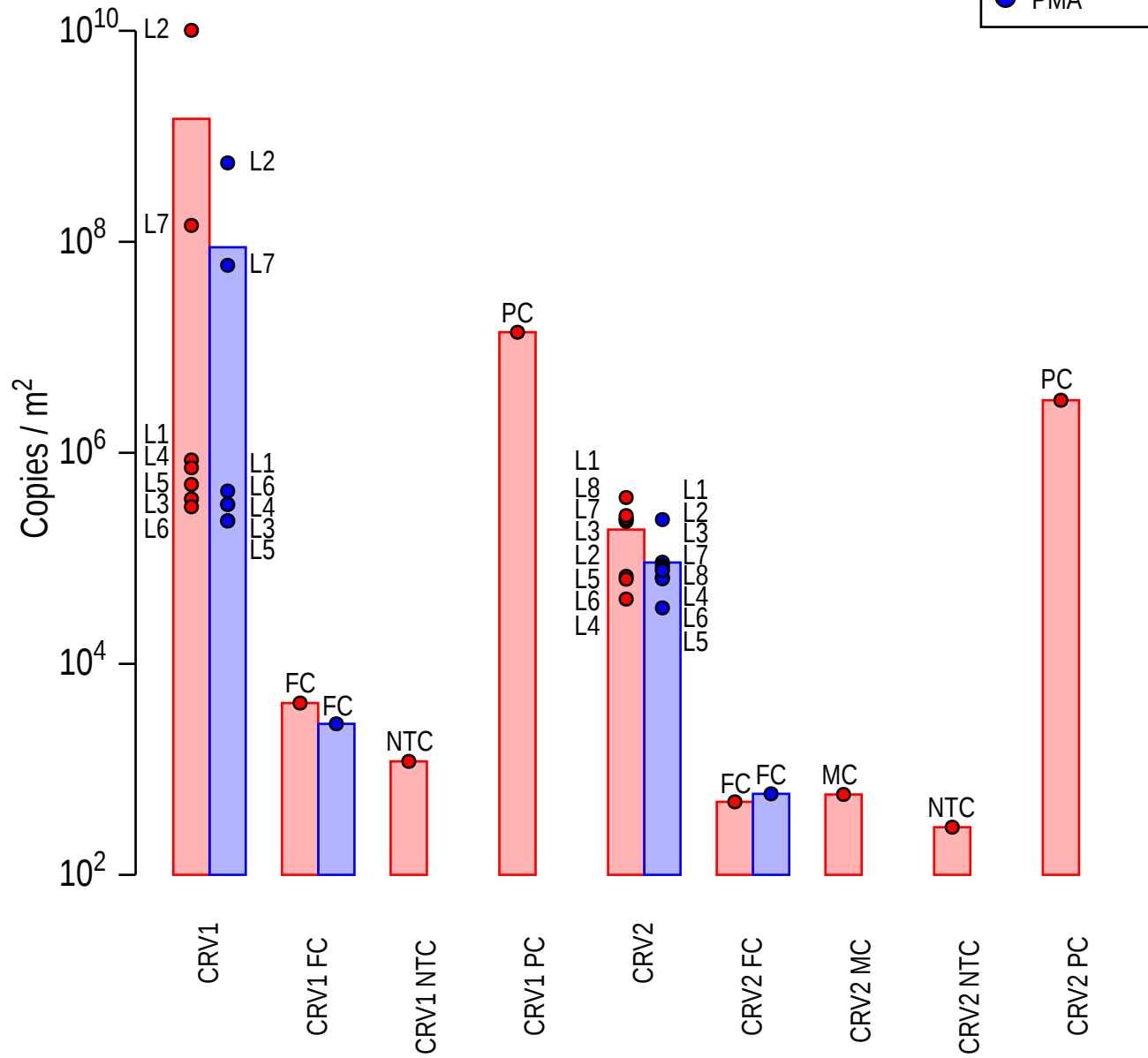

B

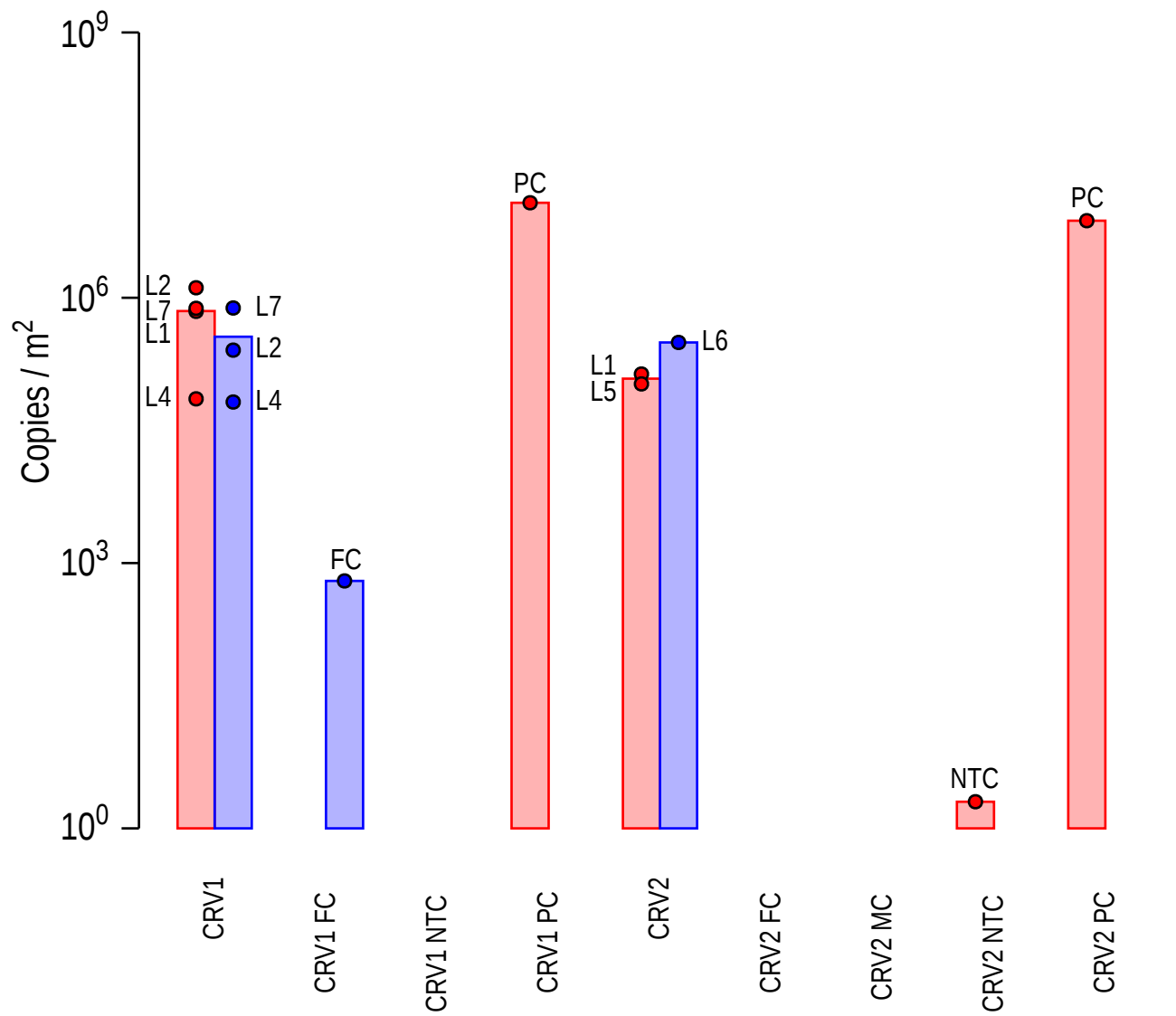

S3

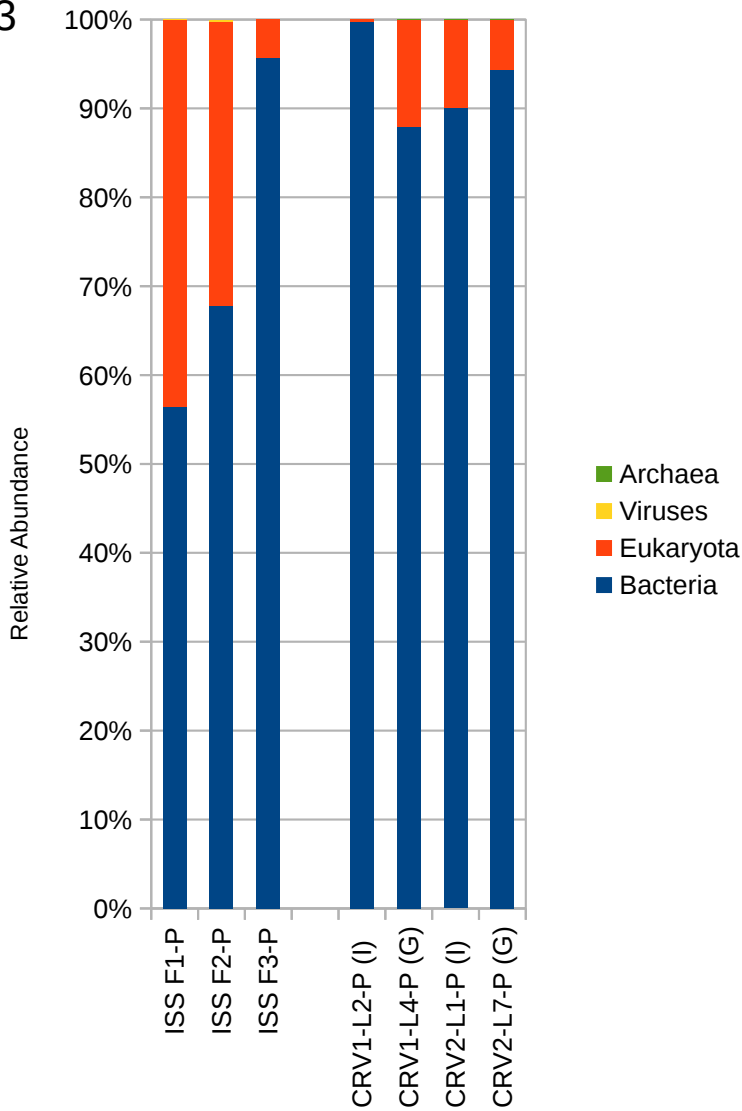

S4

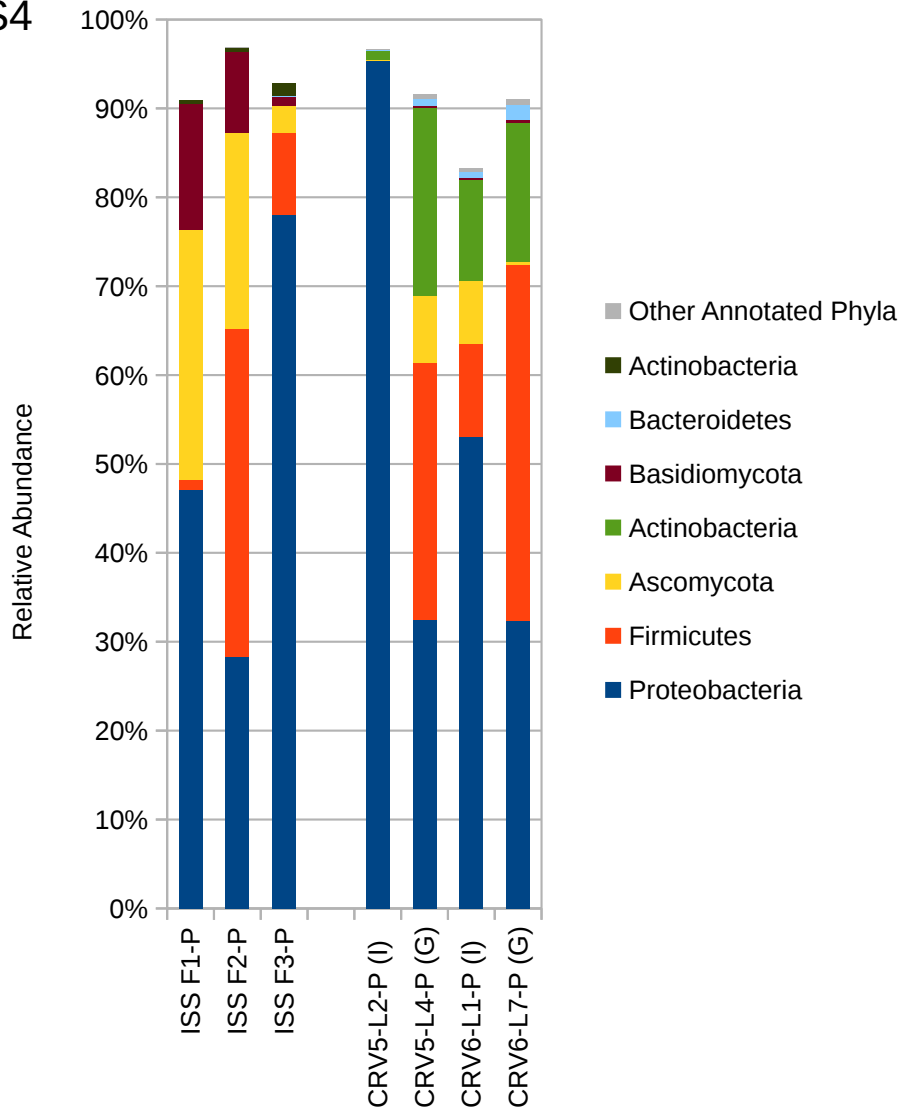

S5

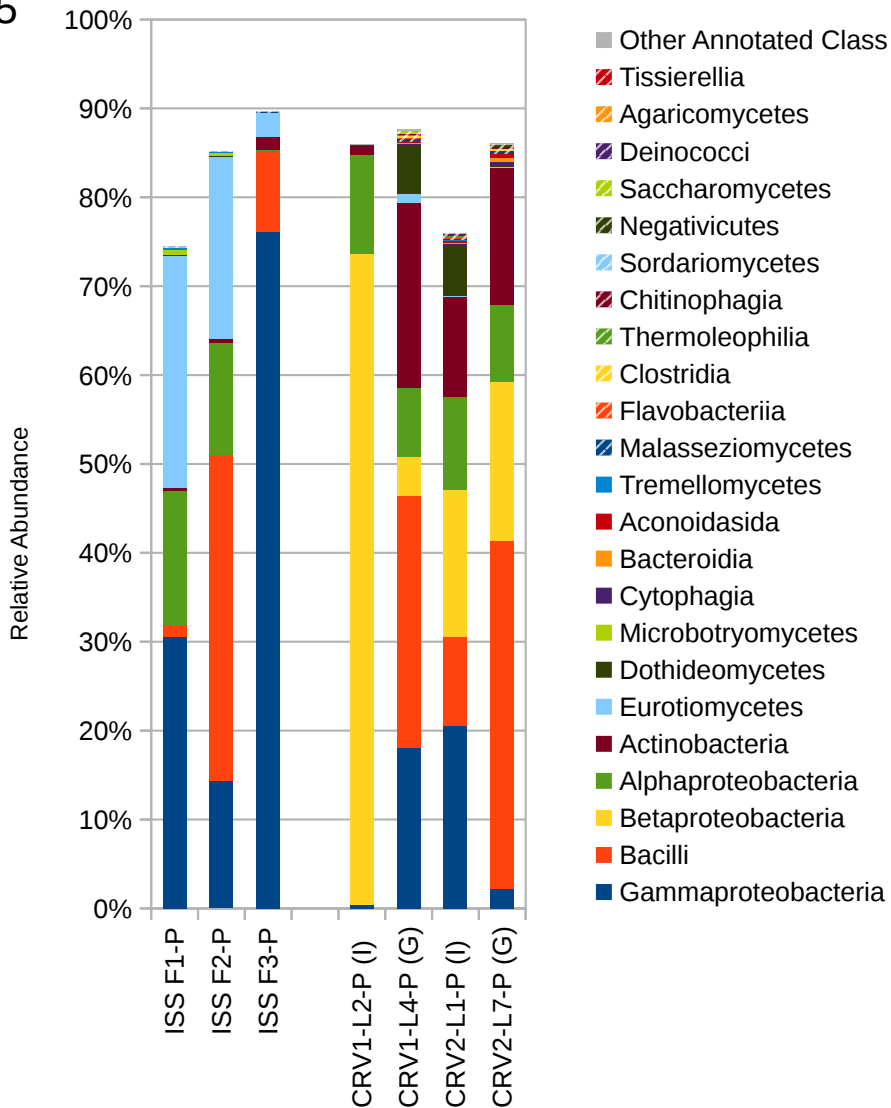

S6

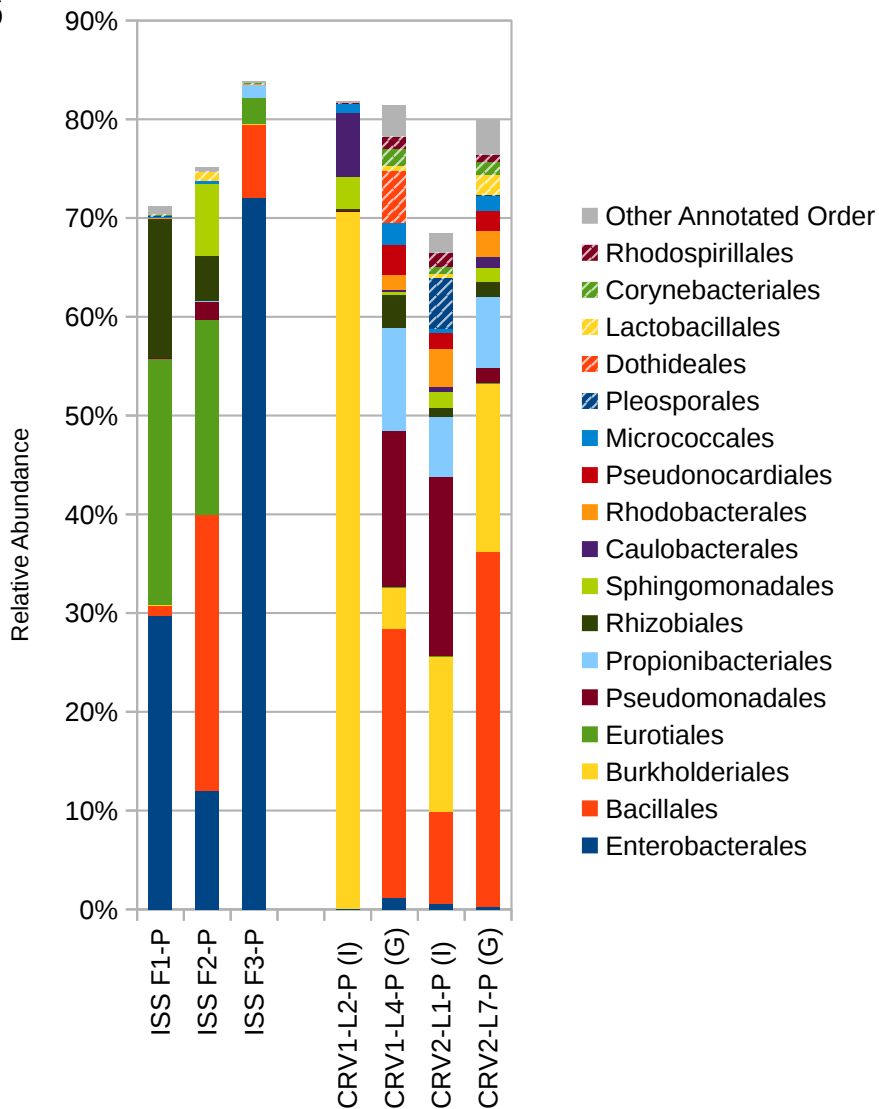

Relative Abundance

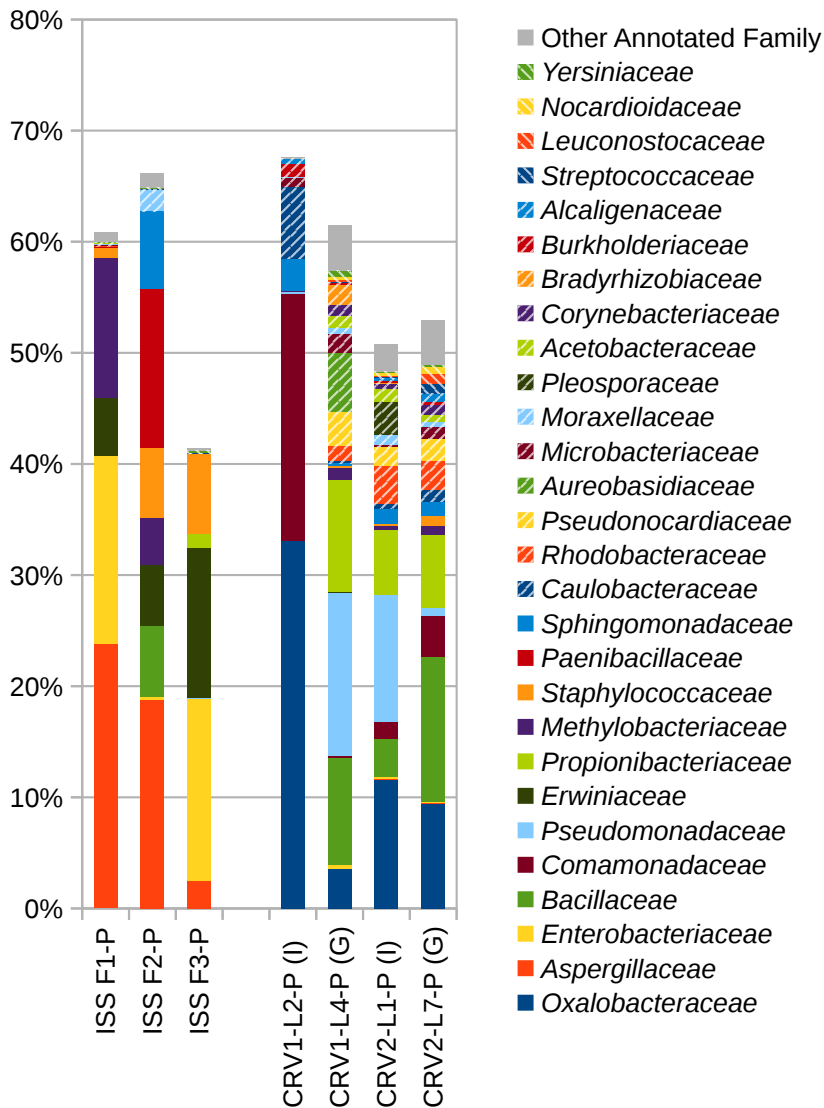

Relative Abundance

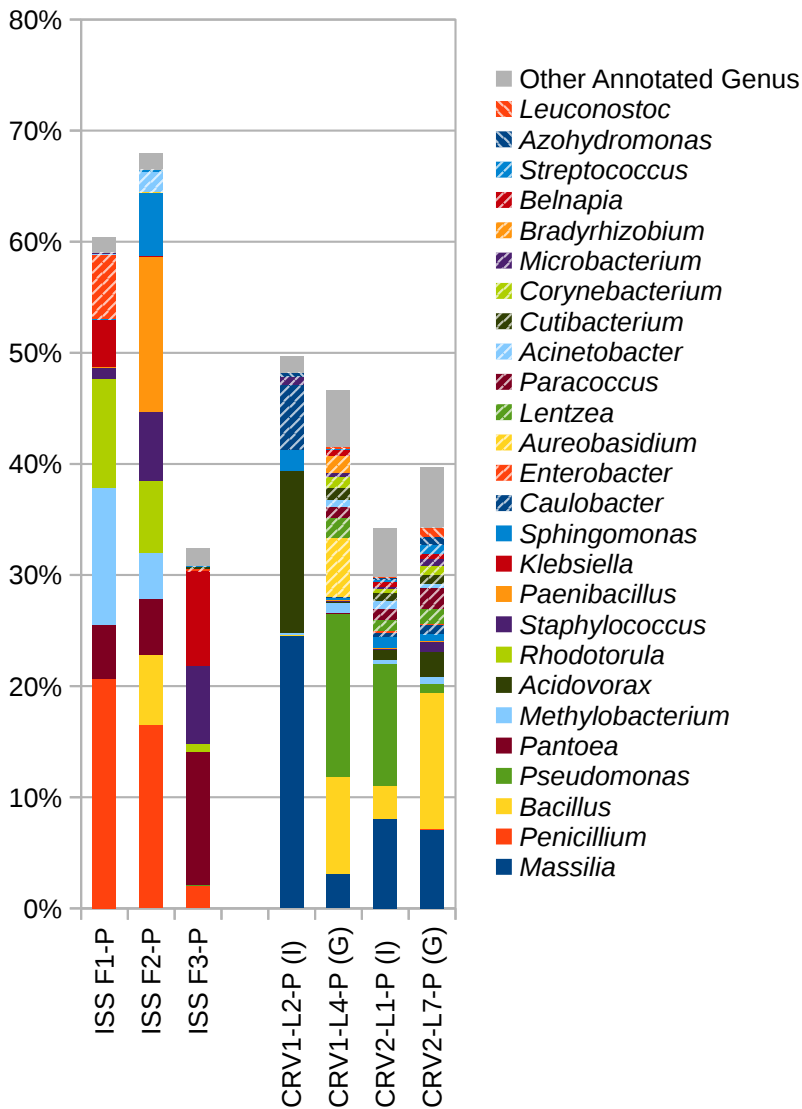

Supplement: Supplementary Figure 1 — Total and Intracellular ATP measured in field control (FC) and surface samples collected from CRV1 and CRV2. [file Data_Sheet_2.pdf]
