# Supplementary material for: Assessing the Risk of Transfer of Microorganisms at the International Space Station Due to Cargo Delivery by Commercial Resupply Vehicles
Source: Front Microbiol. 2020 Nov 6;11:566412. doi: 10.3389/fmicb.2020.566412 (PMC7677455; doi:10.3389/fmicb.2020.566412)
Supplement: Supplementary file 2 [file Data_Sheet_1.pdf]

**Table S1. Identification of CRV strains using Sanger sequencing targeting near full length 16S rRNA gene for bacteria and ITS region for fungi**

| Isolate | Kingdom | Phyla | Nearest neighbor |
|---|---|---|---|
| IG8SW-B2 | Bacteria | Actinobacteria | *Arthrobacter humicola* |
| IG5SW-B5 | Bacteria | Actinobacteria | *Compostimonas suwonensis* |
| IIG8SW-B3 | Bacteria | Actinobacteria | *Curtobacterium oceanosedimentum* |
| IIG8SW-B5 | Bacteria | Actinobacteria | *Curtobacterium oceanosedimentum* |
| IIG8SW-P1 | Bacteria | Actinobacteria | *Curtobacterium oceanosedimentum* |
| IG8SW-B1 | Bacteria | Actinobacteria | *Kocuria turfanensis* |
| IIG3SW-B | Bacteria | Actinobacteria | *Microbacterium marinum* |
| IIG3SW-P1 | Bacteria | Actinobacteria | *Microbacterium natoriense* |
| IIG7SW-B3 | Bacteria | Actinobacteria | *Microbacterium natoriense* |
| IIG7SW-P1 | Bacteria | Actinobacteria | *Microbacterium natoriense* |
| IIG7SW-P2 | Bacteria | Actinobacteria | *Microbacterium natoriense* |
| IG2SW-B5 | Bacteria | Actinobacteria | *Microbacterium oleivorans* |
| IG7SC-B3 | Bacteria | Actinobacteria | *Micrococcus yunnanensis* |
| IG7SC-B5 | Bacteria | Actinobacteria | *Micrococcus yunnanensis* |
| IG7SC-B7 | Bacteria | Actinobacteria | *Micrococcus yunnanensis* |
| IIG1SC-B1 | Bacteria | Actinobacteria | *Micrococcus yunnanensis* |
| IIG3SC-B1 | Bacteria | Actinobacteria | *Micrococcus yunnanensis* |
| IG2SC-B1 | Bacteria | Actinobacteria | *Mycobacterium obuense* |
| IG5SW-B4 | Bacteria | Actinobacteria | *Pseudarthrobacter siccitolerans* |
| IG8SW-B3 | Bacteria | Actinobacteria | *Pseudarthrobacter siccitolerans* |
| IIG1S2-P2 | Bacteria | Actinobacteria | *Rhodococcus defluvii* |
| IIG1SW-P1 | Bacteria | Actinobacteria | *Rhodococcus defluvii* |
| IIG1SW-B3 | Bacteria | Actinobacteria | *Rhodococcus degradans* |
| IG8SW-B4 | Bacteria | Bacteroidetes | *Hymenobacter gelipurpurascens* |
| IIG7SW-B2 | Bacteria | Bacteroidetes | *Hymenobacter monticola* |
| IG9SW-P1 | Bacteria | Firmicutes | *Bacillus pumilus* |
| IG3SW-B1 | Bacteria | Firmicutes | *Bacillus subtilis* |
| IG4SW-B2 | Bacteria | Firmicutes | *Brevibacterium frigoritolerans* |
| IG1SW-B5 | Bacteria | Firmicutes | *Paenibacillus amylolyticus* |
| IG7SC-B2 | Bacteria | Firmicutes | *Staphylococcus epidermidis* |
| IG7SC-B8 | Bacteria | Firmicutes | *Staphylococcus epidermidis* |
| IG7SC-B9 | Bacteria | Firmicutes | *Staphylococcus epidermidis* |
| IG2SW-B2 | Bacteria | Proteobacteria | *Acidovorax wautersii* |
| IG7SC-B1 | Bacteria | Proteobacteria | *Bradyrhizobium elkanii* |
| IG6SW-B3 | Bacteria | Proteobacteria | *Massilia arvi* |
| IG2SW-B4 | Bacteria | Proteobacteria | *Massilia varians* |
| IG5SW-B2 | Bacteria | Proteobacteria | *Massilia varians* |
| IG6SW-B1 | Bacteria | Proteobacteria | *Methylobacterium tardum* |
| IIG7SC-B1 | Bacteria | Proteobacteria | *Sphingomonas asaccharolytica* |
| IG2SW-B1 | Bacteria | Proteobacteria | *Sphingomonas dokdonensis* |
| IG4SW-B1 | Bacteria | Proteobacteria | *Sphingomonas dokdonensis* |
| IG7SW-B1 | Bacteria | Proteobacteria | *Sphingomonas dokdonensis* |
| IG5SW-B1 | Bacteria | Proteobacteria | *Sphingomonas melonis* |
| IIG1SW-B2 | Bacteria | Proteobacteria | *Sphingomonas mucosissima* |
| IIG1SW-B4 | Bacteria | Proteobacteria | *Sphingomonas mucosissima* |

| | | | |
|---|---|---|---|
| IIG1SW-B6 | Bacteria | Proteobacteria | *Sphingomonas mucosissima* |
| IG1SW-B3 | Bacteria | Proteobacteria | *Sphingomonas yunnanensis* |
| IIG8SC-F1 | Fungi | Ascomycota | *Alternaria* sp. |
| IIG1SW-F2 | Fungi | Ascomycota | *Aureobasidium pullulans* |
| IG8SW-F2 | Fungi | Ascomycota | *Cladosporium aphidis* |
| IG7SC-B4 | Fungi | Ascomycota | *Cladosporium* sp. |
| IG2SW-F1 | Fungi | Ascomycota | *Colletotrichum* sp. |
| IG1SW-F3 | Fungi | Ascomycota | *Exophiala xenobiotica* |
| IIG1SC-B2 | Fungi | Ascomycota | *Periconia* sp. |
| IG1SW-F2 | Fungi | Ascomycota | *Phialemoniopsis dimorphosporum* |
| IIG8SW-B1 | Fungi | Ascomycota | *Phitomyces* sp. |
| IG5SW-B3 | Fungi | Ascomycota | *Talaromyces* sp. |
| IIG1SW-F5 | Fungi | Ascomycota | unidentified Ascomycete |
| IIG1SW-B1 | Fungi | Basidiomycota | *Cryptococcus aureus* |
| IIG7SW-F2 | Fungi | Basidiomycota | *Cryptococcus rajasthanensis* |
| IIG7SW-F3 | Fungi | Basidiomycota | *Dioszegia* sp. |
| IIG8SW-B2 | Fungi | Basidiomycota | *Dioszegia* sp. |
| IIG8SW-F1 | Fungi | Basidiomycota | *Hannaella* sp.* |
| IG1SW-B4 | Fungi | Basidiomycota | *Naganishia* sp.* |
| IIG1SW-F1 | Fungi | Basidiomycota | *Papiliotrema pseudoalba* ** |
| IG5SW-F1 | Fungi | Basidiomycota | *Phlebia acerina* |
| IIG7SW-F1 | Fungi | Basidiomycota | *Rhodotorula mucilaginosa* |

* formerly known as *Cryptococcus* sp. (Liu et al. 2015; Wang & Bai 2008)

** formerly known as *Bullera pseudoalba* (Liu et al. 2015)

**Table S2:** Mann-Whitney-Wilcoxon statistics comparing samples collected from ground support equipment surfaces or surfaces internal to the CRV (top), or comparing samples collected during the CRV1 sampling event with samples collected during the CRV2 sampling event (bottom).

| | Internal vs. Ground Support Equipment Surfaces | | | | | | | |
|---|---|---|---|---|---|---|---|---|
| | CRV1 | | | | CRV2 | | | |
| | PMA | | No PMA | | PMA | | No PMA | |
| | W | p-value | W | p-value | W | p-value | W | p-value |
| **Domain** | 43.0 | 0.0996 | 40.0 | 0.0689 | 50.5 | 0.0858 | 74.0 | 0.1148 |
| **Phylum** | 253.5 | 0.1427 | 195.0 | 0.2969 | 131.0 | 0.1497 | 175.0 | 0.0077 |
| **Class** | 573.0 | 0.0154 | 1309.0 | 0.0000 | 417.0 | 0.1018 | 851.0 | 0.0004 |
| **Order** | 2168.5 | 0.0016 | 3743.5 | 0.0001 | 1603.0 | 0.0086 | 3338.0 | 0.0000 |
| **Family** | 6371.0 | 0.0000 | 13455.0 | 0.0000 | 4388.0 | 0.0000 | 10043.0 | 0.0000 |
| **Genus** | 11956.0 | 0.0000 | 25192.0 | 0.0000 | 13686.0 | 0.0000 | 25139.0 | 0.0000 |
| **Species** | 22680.0 | 0.0002 | 36999.0 | 0.0000 | 33967.0 | 0.0155 | 69400.0 | 0.0000 |
| **Metabolism** | 52.0 | 0.0000 | 127.0 | 0.0000 | 532.0 | 0.0000 | 491.0 | 0.0000 |
| **Virulence** | 176.5 | 0.0000 | 413.0 | 0.0000 | 300.0 | 0.0000 | 250.0 | 0.0000 |

| | CRV1 vs. CRV2 | | | | | | | |
|---|---|---|---|---|---|---|---|---|
| | Internal Surface | | | | Ground Support Equipment Surface | | | |
| | PMA | | No PMA | | PMA | | No PMA | |
| | W | p-value | W | p-value | W | p-value | W | p-value |
| **Domain** | 109.0 | 0.2183 | 131.0 | 0.1353 | 111.0 | 0.1817 | 121.0 | 0.0647 |
| **Phylum** | 448.0 | 0.0439 | 462.5 | 0.0230 | 181.5 | 0.6216 | 292.0 | 0.2069 |
| **Class** | 1060.0 | 0.1100 | 22249.0 | 0.0004 | 351.0 | 0.0109 | 581.0 | 0.0000 |
| **Order** | 4314.0 | 0.0275 | 8213.0 | 0.0014 | 1358.5 | 0.0008 | 3419.0 | 0.0000 |
| **Family** | 11140.0 | 0.0014 | 24310.0 | 0.0000 | 2861.0 | 0.0000 | 6037.0 | 0.0000 |
| **Genus** | 24194.0 | 0.0204 | 53416.0 | 0.0000 | 7159.0 | 0.0000 | 12438.0 | 0.0000 |
| **Species** | 54808.0 | 0.1890 | 99639.0 | 0.0918 | 13731.0 | 0.0000 | 22315.0 | 0.0000 |
| **Metabolism** | 1467.0 | 0.0002 | 1853.5 | 0.0000 | 2451.0 | 0.0000 | 2137.0 | 0.0000 |
| **Virulence** | 709.0 | 0.0013 | 1091.5 | 0.0000 | 1718.5 | 0.0000 | 1985.5 | 0.0000 |
